# Cell-Free-Based Thermophilic Biocatalyst for the Synthesis of Amino Acids from One-Carbon Feedstocks

**DOI:** 10.1021/acssynbio.5c00352

**Published:** 2025-10-18

**Authors:** Ray Westenberg, Shaafique Chowdhury, Ryan Cardiff, Kimberly Wennerholm, Alexander S. Beliaev, James M. Carothers, Pamela Peralta-Yahya

**Affiliations:** † 1372 Bioengineering Program, Georgia Institute of Technology, Atlanta, Georgia 30332, United States; ‡ School of Chemical & Biomolecular Engineering, Georgia Institute of Technology, Atlanta, Georgia 30332, United States; § Molecular Engineering & Sciences Institute and Center for Synthetic Biology, 7284 University of Washington, Seattle, Washington 98195, United States; ∥ School of Chemistry and Biochemistry, Georgia Institute of Technology, Atlanta, Georgia 30332, United States; ⊥ Environmental Molecular Sciences Division, 6865 Pacific Northwest National Laboratory, Richland, Washington 99354, United States; # Centre for Agriculture and the Bioeconomy, School of Biological and Environmental Sciences, Queensland University of Technology, Gardens Point Campus, P.O. Box 2434, Brisbane, Queensland 4001, Australia; ¶ Department of Chemical Engineering, University of Washington, Seattle, Washington 98195, United States

**Keywords:** carbon-negative synthesis, chemical bioproduction, cell-free expression, metabolic engineering, thermophilic biocatalyst

## Abstract

Bioproduction from one-carbon compounds, such as formate, is an attractive prospect due to reduced energy requirements and the possibility for using CO_2_ as a sustainable feedstock. Formate-fixing pathways engineered using *Escherichia coli* lysate-based cell-free expression (CFE) biocatalysts have the potential to route 100% of feedstock carbon toward chemical synthesis but are undermined by siphoning of in-pathway metabolites and cofactors by the CFE background metabolism. To address this limitation, we engineer a CFE-based thermophilic multienzyme biocatalyst for the synthesis of serine and glycine from formate, bicarbonate, and ammonia. After expression of the thermophilic formate-to-serine pathway in a one-pot reaction, the mesophilic *E. coli* CFE background machinery is removed by simple heat denaturation, eliminating the siphoning of cofactors, in-pathway metabolites, and products. After bioprocess optimization, including pathway gene expression duration and chemical synthesis temperature, we achieve near stoichiometric conversion of formate and bicarbonate to serine and glycine, reaching 97% of stoichiometric yield. The use of a moderately thermophilic biocatalyst allowed chemical synthesis to take place at mesophilic temperatures, enabling the balance of optimal enzyme activity with minimal metabolite/cofactor thermal degradation. In a fed-batch experiment, the biocatalyst shows sustained chemical synthesis rates for 8 h, paving the way toward a continuous bioprocess. Finally, a sensitivity analysis of cofactor usage revealed that the most expensive cofactors, THF and NADPH, can be reduced by 5-fold without significantly lowering product yields. To the best of our knowledge, this is the first instance of expressing a thermophilic pathway in an *E. coli* lysate-based CFE system to generate a thermophilic biocatalyst for use at mesophilic temperatures. The CFE-based thermophilic formate-to-serine biocatalyst triples the combined serine and glycine yield previously obtained by a CFE-based mesophilic formate-to-serine biocatalyst (30%), and quadruple the yield obtained by a purified enzyme system (22%). Ultimately, this work opens the door to using *E. coli* lysate-based CFE for thermophilic biocatalyst generation to achieve high chemical synthesis yields.

## Graphical abstract

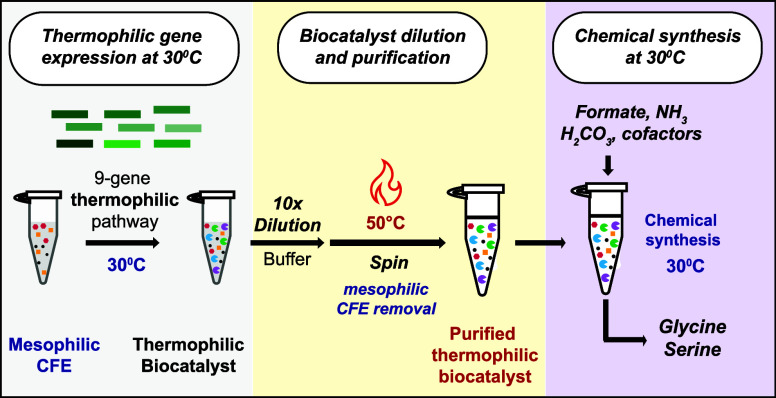

## Introduction

Biological upgrading of CO_2_ or its equivalents—such as the more biologically accessible formate—directly into value-added chemicals has the potential to remove CO_2_ from the environment at a lower energy and cost than starting from other feedstocks.^1^ Limitations with natural carbon fixing organisms, including their slow growth and low carbon fixation efficiency,^2^ have led to the implementation of natural and synthetic carbon fixation pathways in biotechnology-friendly chassis, such as *Escherichia coli* ^3,4^ and *Saccharomyces cerevisiae*,^5,6^ to leverage their fast growth rate and the extensive body of knowledge about their metabolic pathway optimization.^7^ Among carbon fixation pathways, the tetrahydrofolate (THF)-dependent formate fixation/reductive glycine synthesis (THF/rGS) pathway is of particular interest, given its low energy requirements (2 ATP, 3 NAD­(P)­H), thermodynamic favorability (Δ*G* = −4.9 kJ/mol from formate to glycine), and minimal enzyme requirements (9 enzymes). To date, the THF/rGS pathway has been implemented in *Escherichia coli* ^1,8–10^ and yeast^6,11^ to support cellular growth and has enabled 10% yield of lactate from formate.^12^

Recently, the THF/rGS pathway has been implemented in an *E. coli* lysate-based cell-free expression (CFE) system to generate a 10-enzyme biocatalyst for the carbon-negative synthesis of serine and glycine from formate.^13^ Briefly, CFE systems can be generated using either (1) cell lysate, i.e., unpurified cell extract containing transcription/translation machinery as well as enzymes involved in primary metabolism, or (2) purified cell machinery (PURE), i.e., the purified 36 enzymes and ribosomes involved in transcription/translation.^14^ The enthusiasm around engineering CFE systems for chemical synthesis is because they are nonliving; consequently, carbon feedstock is not needed for cell growth and maintenance; thus, they are capable of potentially routing 100% of carbon feedstock to chemical synthesis. The PURE system has very low CFE background metabolism; however, it is more time-consuming and expensive to generate ($600–$2000/L^15^), likely rendering it cost-prohibitive for bioindustrial applications. On the other hand, lysate-based CFE systems have a lower production cost ($90/L^16^) and can be rapidly scaled up; however, the significant CFE background metabolism (or CFE background machinery) siphons cofactors and intermediates away from the desired metabolic pathway, reducing the desired product yields. Specifically, the lysate-based CFE-based mesophilic THF/rGS biocatalyst resulted in a 30% yield of serine and glycine, with intermediates and cofactors siphoned by CFE background metabolism**—**in particular NAD­(P)­H-consuming pathways^13,17^ **—**which was attributed as one of the major process limitations to achieving higher product yields.

Synthetic biology and bioprocess engineering strategies can be implemented to reduce the CFE background metabolism. Synthetic biology strategies include (1) knocking out competing metabolic pathways in the microbes used to generate the CFE cell lysate^18–21^ and (2) adding inhibitors directly to the CFE to block competing pathways.^17,21^ Knocking out competing pathways in the *E. coli* used to make the CFE cell lysate resulted in more than a 2-fold increase in protein synthesis yields.^18^ Although there is no concrete evidence that this strategy will result in higher chemical synthesis yields in the context of a CFE-based biocatalyst, it is likely to do so given prior work around chemical synthesis in crude cell lysates.^22^ For example, knocking out competing pathways in the *E. coli* used to make the crude cell lysate led to an 85% reduction in the use of ATP for the synthesis of dihydroxyacetone phosphate.^20^ Direct addition of inhibitors to the CFE system to block the TCA cycle has resulted in a 20% increase in malate synthesis.^17^ Looking ahead, the use of pathway enzymes engineered to use artificial cofactors^23^ is also likely to improve product yields. Bioprocess engineering strategies shown to reduce the effect of CFE background metabolism include (1) tagging key genes for affinity purification removal before generating the CFE^24,25^ and (2) diluting the CFE to minimize background metabolism.^13,17^ In crude cell lysates, tagging and removing key enzymes has led to a 4-fold improvement in ethanol synthesis^24^ and 40-fold improvement in pyruvate synthesis,^25^ suggesting it could improve yields for a CFE-based biocatalyst. Nevertheless, this strategy is laborious, requiring tagging of the lysate-source genome, followed by bead-based purification. Although CFE dilution reduces background metabolism, at 10× dilution, siphoning of NADH by the background CFE metabolism was still significant.^13^

A common strategy to rapidly and inexpensively purify enzymes^16^ is the expression of thermophilic genes in *E. coli* to generate enzymes with a temperature optimum between 50° and 90 °C, followed by cell lysis and heat denaturation to remove the native *E. coli* proteins that precipitate between 45° and 50 °C.^26^ This heat denaturation strategy has been used to produce thermophilic multienzyme biocatalysts for the synthesis of pyruvate (9 enzymes),^27^ glutathione (2 enzymes),^28^ acetoin (2 enzymes),^29^ fructose 1,6-diphosphate (4 enzymes),^30^ myo-inositol (4 enzymes^31^ and 11 enzymes^32^), glucaric acid (3 thermostable enzymes),^33^ and cysteine (11 enzymes).^34^ In all of these cases, chemical synthesis has taken place at the optimal temperature of the biocatalyst, 70°–90 °C. A key challenge of performing chemical synthesis at thermophilic temperatures is the instability of cofactors and metabolites.^32,35,36^ Of note, not all thermophilic proteins will fold correctly at mesophilic temperatures or in mesophilic organisms such as *E. coli*.^37^

An analogous strategy in CFE involves expression of a thermophilic pathway in an *E. coli* lysate-based CFE to generate the thermophilic biocatalyst followed by heat denaturation to remove the *E. coli* proteins, i.e., the CFE background machinery. Such a strategy would allow the rapid prototyping of thermophilic biocatalysts in the absence of CFE background metabolism. Additionally, the lack of a cell membrane and the ability of CFE to withstand toxic compounds also open the door to complementary bioprocess optimization solutions, such as direct addition of inhibitors to improve chemical synthesis. Further, available tools for codon optimization of thermophilic genes to match the *E. coli* codon usage together with inexpensive DNA synthesis improve the feasibility of prototyping thermophilic genes for biocatalyst generation. An important consideration is that not all thermophilic genes will express in a mesophilic system, thus, thermophilic CFE platforms have been developed for this purpose.^35,38–40^ However, the expression of thermophilic genes in a thermophilic CFE system would not facilitate the rapid removal of the CFE machinery by simply increasing the reaction temperature.

Here, we engineer a 9-enzyme CFE-based thermophilic biocatalyst for the synthesis of serine and glycine from formate, bicarbonate, and ammonia (Figure 1A). The biocatalyst captures 3 total CO_2_ equivalents with 2 CO_2_ equivalents (1 formate and 1 bicarbonate) captured per glycine molecule produced and an additional 1 CO_2_ equivalent (1 formate) per serine molecule produced (Figure 1B). The use of a thermophilic biocatalyst enables the removal of the mesophilic *E. coli* CFE background machinery after gene expression, impeding the siphoning of intermediates and cofactors away from the thermophilic biocatalyst. The 9-gene THF/rGS thermophilic pathway is generated in a one-pot reaction using *E. coli* lysate-based CFE. Since previous efforts have focused on reconstituting the mesophilic THF/rGS pathway,^1,8,9,12,13,41^ this is the first generation of a thermophilic THF/rGS pathway. To balance enzyme activity with minimal cofactor degradation, chemical synthesis was performed at mesophilic temperatures (30 °C) rather than thermophilic temperatures (over 50 °C). Via bioprocess optimizations, the CFE-based thermophilic biocatalyst achieves near stoichiometric yield of serine and glycine (97%) from one-carbon feedstocks using a 20 h multigene expression step and a 4 h chemical synthesis step. This yield is 3-fold higher than that using a CFE-based mesophilic formate-to-serine biocatalyst (30%),^13^ 4-fold higher than that using a purified enzyme system (22%),^42^ and almost 8-fold higher than previous microbial engineering efforts to produce lactate from formate in *E. coli*.^12^ This study is therefore the highest reported conversion of CO_2_ equivalents into amino acids compared to any previous *E. coli* whole cell, purified, or CFE-based biocatalyst.

**Figure 1:**
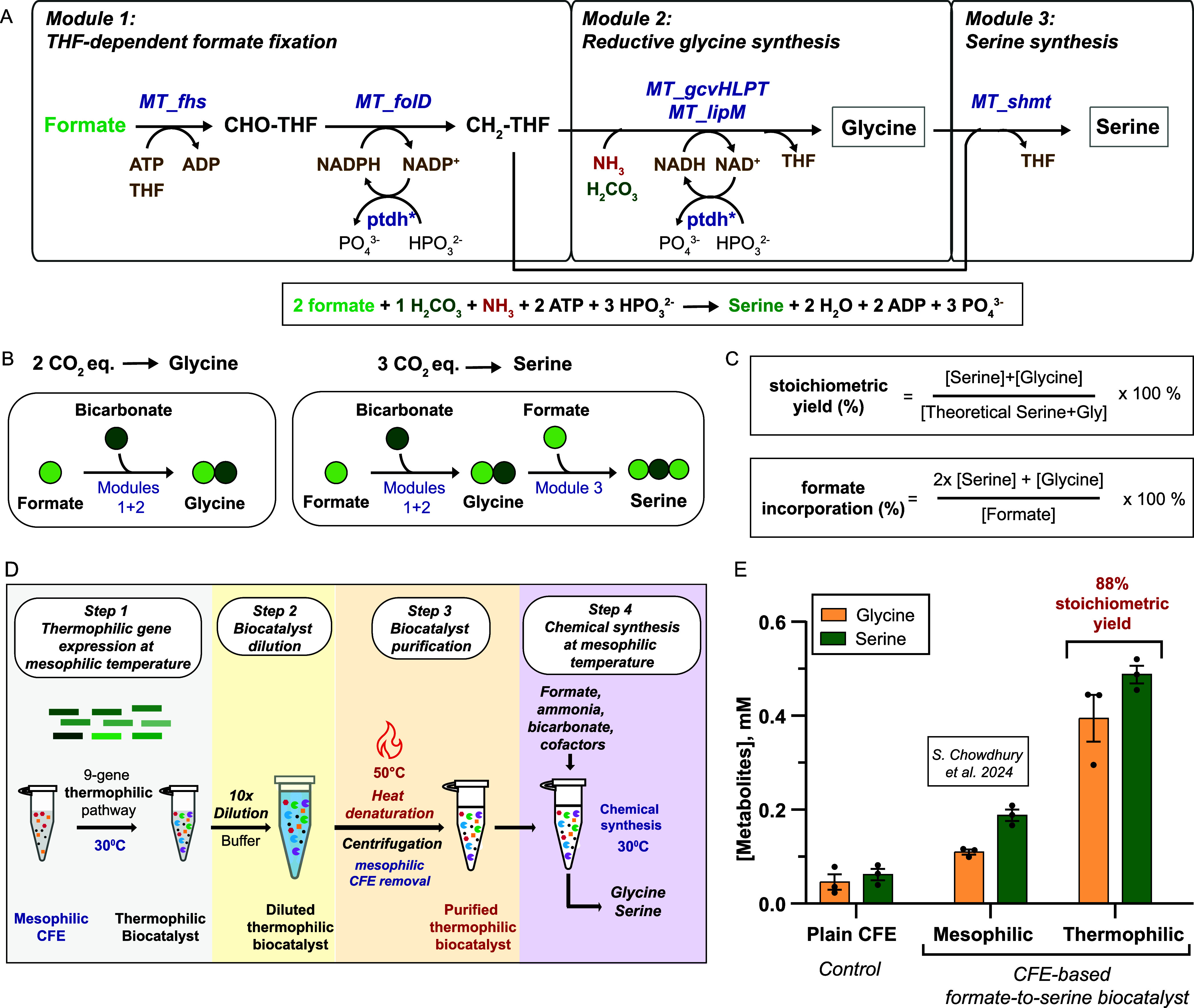
Cell-free expression (CFE)-based thermophilic biocatalyst for the carbon-negative synthesis of serine and glycine from formate, bicarbonate, and ammonia. (A) CFE-based thermophilic formate-to-serine biocatalyst. Enzyme (blue): *MT_fhs*, *Moorella thermoacetica* formate-tetrahydrofolate ligase; *MT_folD*, *M. thermoacetica* bifunctional protein performing the activities of methenyltetrahydrofolate cyclohydrolase and methylenetetrahydrofolate dehydrogenase; *MT_gcvHLPT*, *M. thermoacetica* glycine cleavage system H, L, P, and T proteins; *MT_lipM*, *M. thermoacetica* octanoyltransferase; *MT_shmt*, *M. thermoacetica* serine hydroxymethyltransferase; ptdh*, *Pseudomonas stutzeri* phosphite dehydrogenase mutant. Substrates (green, red): H_2_CO_3_, bicarbonate, NH_3_, ammonia. Metabolites (gray): CHO–THF, 10-formyltetrahydrofolate, CH_2_–THF, 5,10-methylenetetrahydrofolate. Cofactors (brown): THF, tetrahydrofolate, ATP, NAD­(P)­H. (B) Cartoon representation of carbons (formate, bicarbonate) incorporated into glycine and serine. (C) Equations for stoichiometric yield and formate incorporation used in this study. (D) CFE-based thermophilic biocatalyst bioprocess. Step 1: One-pot expression of pathway genes for biocatalyst generation. Step 2: Biocatalyst dilution. Step 3: Heat denaturation of the mesophilic CFE machinery followed by debris removal via centrifugation. Step 4: Thermophilic biocatalysis performs chemical synthesis at mesophilic temperatures (30 °C). (D) Synthesis of serine and glycine from formate, bicarbonate, and ammonia obtained via a CFE-based mesophilic formate-to-serine biocatalyst (Chowdhury et al. 2024 ^13^) or the CFE-based thermophilic formate-to-serine biocatalyst (this work). Reaction conditions: Gene expression: 30 °C, 16 h; dilution: 10×; heat denaturation: 50 °C, 10 min; chemical synthesis: 30 °C, 4 h; supplementation: 2 mM CH_2_O_2_, 2 mM THF, 1 mM H_2_CO_3_, 1 mM NH_3_, 2 mM ATP, 1 mM NADH, 2 mM NADPH, 5 mM Na_2_HPO_3_. The bars represent the mean ± standard error of the mean (SEM), *n* = 3, ****p < 0.0001. Data were analyzed using a two-way ANOVA followed by a multiple comparisons test via the Tukey method. Calculations can be found in Table S2.

## Results

### Formate-to-Serine Biocatalyst

For engineering purposes, we split the formate-to-serine pathway into three modules (Figure 1A). In module 1, THF-dependent formate fixation, formate (CH_2_O_2_) attaches to THF to become the C1 donor CH_2_–THF. In module 2, reductive glycine synthesis, CH_2_–THF combines with bicarbonate (H_2_CO_3_) and ammonia (NH_3_) to generate glycine. In module 3, the serine synthesis, glycine combines with a second CH_2_–THF to generate serine. Modules 2 and 3 recycle THF. Regeneration of NAD­(P)H is achieved via a *Pseudomonas stutzeri* phosphite dehydrogenase mutant.^43^ The cofactor ATP is not recycled in the system.

Based on pathway stoichiometry, 1 mol CH_2_O_2_, 1 mol H_2_CO_3_ (2 CO_2_ equivalents), and 1 mol NH_3_ are needed per mol of glycine generated, while an additional mol of CH_2_O_2_ is needed per mole of serine produced (Figure 1B). To calculate yields of the system, all our experiments were conducted at stoichiometric substrate concentrations, that is, 2 mM CH_2_O_2_, 1 mM H_2_CO_3_, and 1 mM NH_3_, which could result in a maximum concentration of 1 mM combined glycine and serine. Thus, the stoichiometric yield is defined as the actual concentration of serine and glycine divided by the theoretical maximum concentration of serine and glycine produced (1 mM) (Figure 1C and Table S2). We were also interested in determining the percent of formate incorporated into amino acids. This is calculated as two times the serine concentration (2 CH_2_O_2_ per serine generated) plus the glycine concentration divided by the concentration of fed formate (Figure 1C and Table S3).

### Cell-Free Expression-Based Thermophilic Biocatalyst Process Strategy

Removal of the CFE background machinery should eliminate siphoning of metabolic intermediates and cofactors away from the formate-to-serine biocatalyst, thus achieving a close to 100% product yield. Figure 1D shows the envisioned CFE-based thermophilic biocatalyst process. Step 1, gene expression, involves the one-pot expression of the thermophilic formate-to-serine pathway genes in an *E. coli* lysate-based CFE at 30 °C. During the gene expression step, optimal *E. coli* lysate-based CFE transcription/translation conditions^44^ in terms of temperature, cell lysate, energy molecules, cofactors, and buffer are set. In Step 2, biocatalyst dilution, the thermophilic biocatalyst is diluted 10-fold using buffer to increase substrate loading and reach higher product levels. Step 3, biocatalyst purification, entails removal of the mesophilic *E. coli* CFE background machinery by heat denaturation. The precipitated *E. coli* proteins are separated via centrifugation, leaving the thermophilic biocatalyst in the solution phase. In Step 4, chemical synthesis, to the solution phase containing the thermophilic biocatalyst, substrate and cofactors are added and chemical synthesis is run at mesophilic temperatures (30 °C). Use of mesophilic temperatures limits thermal degradation of cofactors, including ATP, NAD^+^, NADH,^32,36,45^ and THF^46,47^ that would have occurred at higher temperatures, thus bypassing a major obstacle when using thermophilic biocatalysts.

### CFE-Based Thermophilic Formate-to-Serine Biocatalyst Design

Due to previous challenges with thermal degradation of cofactors at hyperthermophilic temperatures (70°–90 °C),^32,36,45^ we sought to design a thermophilic formate-to-serine biocatalyst that would withstand heat denaturation of the *E. coli*-based CFE machinery at 45°–50 °C^26^ yet have high activity during the chemical synthesis step performed at mesophilic temperatures (30 °C). Hyperthermophilic enzymes often have greatly reduced activities at lower temperatures,^31^ experiencing over 80% loss of activity below 60 °C.^48^ Thus, we focused on identifying thermophilic enzymes with experimental data confirming optimal activity at moderately thermophilic conditions (50–70 °C).^49^ Such enzymes, we hypothesized, would allow the balance of optimal enzyme activity with minimal metabolite thermal degradation at mesophilic chemical synthesis temperatures.

Despite the sparse kinetic data on thermophilic enzymes,^49^ activity for module 1 enzymes is available for the moderately thermophilic *Moorella thermoacetica* (formerly named *Clostridium thermoaceticum*).^50–53^ *M. thermoacetica* is an anaerobic organism with an optimal growth temperature of 55–60 °C,^54^ naturally performs THF-dependent CO_2_ fixation,^55^ and is a well-studied thermophile, having been the gene source for the engineering of a thermophilic ethanol fermentation pathway.^56^

The thermophilic module 1 is composed of *M. thermoacetica* formate-tetrahydrofolate ligase (Fhs) and the bifunctional NADP^+^-dependent methenyltetrahydrofolate cyclohydrolase/methylenetetrahydrofolate dehydrogenase (FolD). Compared to the mesophilic *Methylorubrum extorquens* *AM1* THF-dependent formate fixation pathway, which has been widely used to reconstitute the THF pathway in mesophilic biotechnology amenable organisms,^1,4,8,9,12,13,41^ the activity of *M. thermoacetica* FolD is performed by two enzymes, *M. extorquens* methenyltetrahydrofolate cyclohydrolase (Fch) and the NADP^+^-methylenetetrahydrofolate dehydrogenase (MtdA). In the *M. extorquens* THF pathway, MtdA is the rate limiting step due to its high reversibility, requiring NADP^+^ recycling to drive the reaction forward.^13^ Kinetic data for Fhs and FolD suggest that the *M. thermoacetica* enzymes are faster than the equivalents from *M. extorquens* (Table S1).

There are no kinetic data for thermophilic enzymes in the reductive glycine pathway (rGS, module 2). Therefore, we used rGS enzymes from the same organism, *M. thermoacetica*, as using enzymes from the same organism supports recapitulation of protein–protein interactions important for protein complex formation.^57^ Briefly, *M. thermoacetica* rGS genes were identified using automated gene predictions from the BioCyc genome database,^58^ as derived from protein homology. BLAST protein analyses were performed to compare the thermophilic protein sequences with their mesophilic homologues. Although the similarity scores were relatively low for most enzymes (Table S1), the statistical significance of the protein alignments was high. Taken together, the thermophilic module 2 is composed of *M. thermoacetica* glycine cleavage system proteins H, L, P, and T (GcvHLPT) and lipoyl­(octanoyl) transferase (LipM). Of note, mesophilic rGS systems have relied on the *E. coli* rGS pathway, which uses lipoate-protein ligase (LplA, EC 6.3.1.20) to perform the sequential reactions of ATP transfer to lipoic acid to generate lipoate-AMP, followed by lipoate transfer to GcvH^41^. In *M. thermoacetica*, a general transferase, LipM, is used to perform both reactions. Finally, the thermophilic module 3 is composed of *M. thermoacetica* serine hydroxymethyltransferase (Shmt). Recycling of NAD­(P)H was performed by a previously engineered thermostable *P. stutzeri* phosphite dehydrogenase (ptdh*),^43^ which has been used for cofactor recycling in CFE.^13^

### Evaluation of the CFE-Based Thermophilic Formate-to-Serine Biocatalyst

The CFE-based mesophilic formate-to-serine biocatalyst achieved a 30% stoichiometric yield using a bioprocess composed of a 16 h gene expression step, 10-fold biocatalyst dilution, and a 4 h chemical synthesis step.^13^ Like in the mesophilic system, in the CFE-based thermophilic biocatalyst, the genes were expressed from a P_T70_ promoter using linear DNA. The formate-to-serine pathway gene ratios resulting in maximal glycine/serine synthesis in the mesophilic system (*gcvHLPT*/*lplA* = 192:2:1:4:2 and *ftl*/*fch*/*mdta*/*shmt*/ptdh* = 3:3:3:3:3)^13^ were used in the thermophilic system. Using the same bioprocess parameters plus heat denaturation to remove the mesophilic CFE background machinery prior to chemical synthesis, the CFE-based thermophilic biocatalyst achieved 88% of the stoichiometric yield of combined serine and glycine from formate and bicarbonate (0.49 mM ± 0.02 mM serine and 0.39 mM ± 0.05 mM glycine), with 68.5% formate incorporation (Figure 1E, Tables S2 and S3). Formate-derived CH_2_–THF is pulled into both module 2 to synthesize glycine (via incorporation of H_2_CO_3_) and module 3 to convert glycine into serine. The remaining ∼30% formate may be in the form of formate or CH_2_–THF, but it has not been combined with glycine to form serine. Taken together, the CFE-based thermophilic formate-to-serine biocatalyst and the outlined bioprocess nearly triple the combined serine and glycine yields achieved by the mesophilic system.^13^

### Impact of the Mesophilic CFE Machinery on Amino Acid Yields

The drastic improvement in amino acid synthesis when using the CFE-based thermophilic biocatalyst coupled to the heat-denaturing treatment led us to investigate the role of the CFE background machinery on amino acid yields. As shown in Figure 2A, we measured the yield of serine and glycine from formate in the presence and absence (due to heat denaturation treatment) of the CFE background machinery over a period of 12 h. For up to 4 h of chemical synthesis, there is no difference in the synthesis of glycine or serine in the presence or absence of the CFE machinery. The chemical synthesis for both serine and glycine peaks at 4 h, reaching 0.39 ± 0.05 mM glycine and 0.49 ± 0.02 mM serine in the absence of the CFE machinery and 0.32 ± 0.01 mM glycine and 0.44 ± 0.04 mM serine in the presence of it. Starting at 8 h, however, the glycine concentration in the presence of the CFE machinery starts to drop while it remains constant in the absence of the CFE machinery. Most dramatically, after 8 h of chemical synthesis, the glycine concentration in the presence of the CFE machinery is 33% lower than in the absence of it. There is a similar trend in the synthesis of serine. After 12 h of chemical synthesis, the serine concentration is 53% lower in the presence of the CFE machinery versus in the absence of it. Given that heat denaturation is the only difference between treatments, the CFE background machinery must consume the serine and glycine synthesized by the biocatalyst from formate, thus reducing the overall chemical yield.

**Figure 2:**
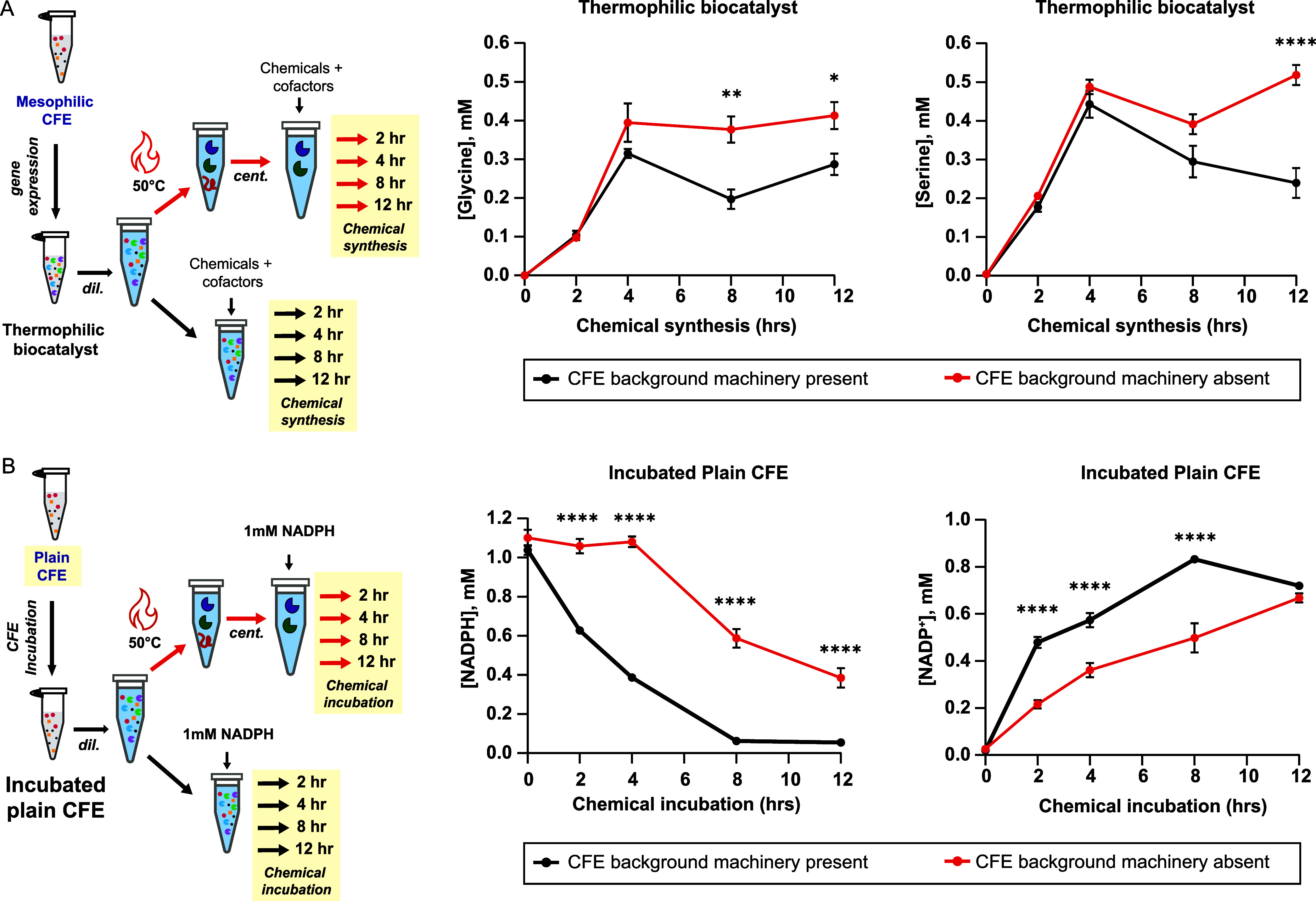
Impact of mesophilic CFE background machinery on amino acid yield and cofactor consumption. (A) Synthesis of serine and glycine from formate, bicarbonate, and ammonia by the thermophilic formate-to-serine biocatalyst in the presence (black) and absence via heat denaturation (red) of the mesophilic CFE background machinery. Left: Process schematic. Right: Glycine or serine concentration as a function of chemical synthesis time. Reaction conditions: Gene expression: 30 °C, 16 h; dilution: 10×; heat denaturation: 50 °C, 10 min or none; chemical synthesis: 30 °C, 2–12 h; supplementation: 2 mM CH_2_O_2_, 2 mM THF, 1 mM H_2_CO_3_, 1 mM NH_3_, 2 mM ATP, 1 mM NADH, 2 mM NADPH, 5 mM Na_2_HPO_3_. Data at the *t* = 0 h time point were taken from the Fmoc glycine and Fmoc serine standard curves using plain CFE (no genes expressed) with Tris buffer and chemical denaturant. Data points represent the mean ± standard error of the mean (SEM), *n* = 3, with *p < 0.05, **p < 0.005, ****p < 0.0001 as a comparison of mesophilic CFE machinery absent (red) versus mesophilic CFE machinery present (black) at the same chemical synthesis time. Data were analyzed using a two-way ANOVA followed by a multiple comparisons test via the Šídák method. (B) Consumption of NADPH incubated in plain CFE in the presence (black) and absence via heat denaturation (red) of the mesophilic CFE background machinery. Reaction conditions: Gene expression: 30 °C, 16 h; dilution: 10×; heat denaturation: 50 °C, 10 min or none; chemical incubation: 30 °C, 2–12 h; supplementation: 1 mM NADPH. Left: Process schematic. Right: NADPH or NADP^+^ concentration as a function of chemical incubation time. Data points represent mean ± SEM, *n* = 3, with ****p < 0.0001 as a comparison of mesophilic CFE background machinery absent (red) versus mesophilic CFE background machinery present (black) at the same chemical incubation time. Data were analyzed using a two-way ANOVA followed by a multiple comparisons test via the Šídák method.

### Impact of the Mesophilic CFE Machinery on Cofactor Availability

Previous work has shown that the CFE background machinery siphons NADH.^13^ Thus, we investigated the effect of the CFE machinery on NADPH concentration as two NADPH equivalents are used per serine synthesized. Using plain CFE (i.e., CFE expressing no genes) incubated at 30 °C for 16 h to mimic the gene expression step and supplemented with 1 mM NADPH, we measured NADPH concentrations in the presence and absence (due to heat-denatured treatment) of the CFE background machinery over a 12 h period. In the presence of the CFE background machinery, close to 40% of the NADPH is consumed in the first 2 h, reaching almost 100% consumption after 8 h (Figure 2B). In the absence of the CFE background machinery, NADPH concentration is unchanged for the first 4 h (1.08 ± 0.03 mM). However, NADPH concentration drops 46% between 4 and 8 h followed by a 34% drop between 8 and 12 h. The halving of the NAPDH concentration after 8 h can be explained by the thermal degradation of NADPH, which has a half-life of ∼8.6 h at pH 7 at 30 °C.^59^

Regarding the NADP^+^ concentration, in the presence of the CFE background machinery, there is an exponential increase in NADP^+^ reaching 0.83 ± 0.01 mM after 8 h and plateauing thereafter. In the absence of the CFE machinery, the NADP^+^ concentration increases linearly, reaching 0.67 ± 0.02 mM after 12 h. The increase in NADP^+^ concentration between 0 and 4 h in the absence of the CFE background machinery is surprising as there is no decrease in the NADPH concentration during the same time period. We hypothesize that the 36% increase in NADP^+^ over the first 4 h may be due to some of the CFE background machinery not being completely removed upon heat denaturation, since some proteins can remain partially soluble in their unfolded states.^60,61^ Unfolded *E. coli* proteins in the supernatant may refold and regain activity upon returning to mesophilic temperatures, reconstituting some of the CFE machinery components. Indeed, we observe that although most of the proteins precipitate upon heat denaturation, some proteins are still precipitated out by a subsequent chemical denaturation step (Figure S1). Taken together, the heat denaturation step removes sufficient CFE background machinery to stop NADPH consumption for the first 4 h, which represents the time required by the thermophilic biocatalyst to achieve close to stoichiometric yields of serine and glycine from formate.

### Biosynthetic Contribution of Enzymes in the THF-Dependent Formate Fixation Module

The presence of the CFE background machinery does not reduce the glycine or serine concentration obtained by the thermophilic biocatalyst up to the 4 h chemical synthesis mark. Thus, we could compare head-to-head the efficiency of the mesophilic and thermophilic THF/rGS biocatalyst using a 4 h chemical synthesis step in the presence of the CFE background machinery. In particular, we were curious about comparing the efficiency of the THF-dependent formate fixation (module 1, Figure 3A), as the mesophilic system relies on two enzymes (*M. extorquens* Fch and MdtA), whereas the thermophilic system uses a single bifunctional enzyme (*M. thermoacetica* FolD). Given the chemical instability of CH_2_–THF (Figure S2), we perform the module 1 experiments using a 30 min chemical synthesis step.

**Figure 3:**
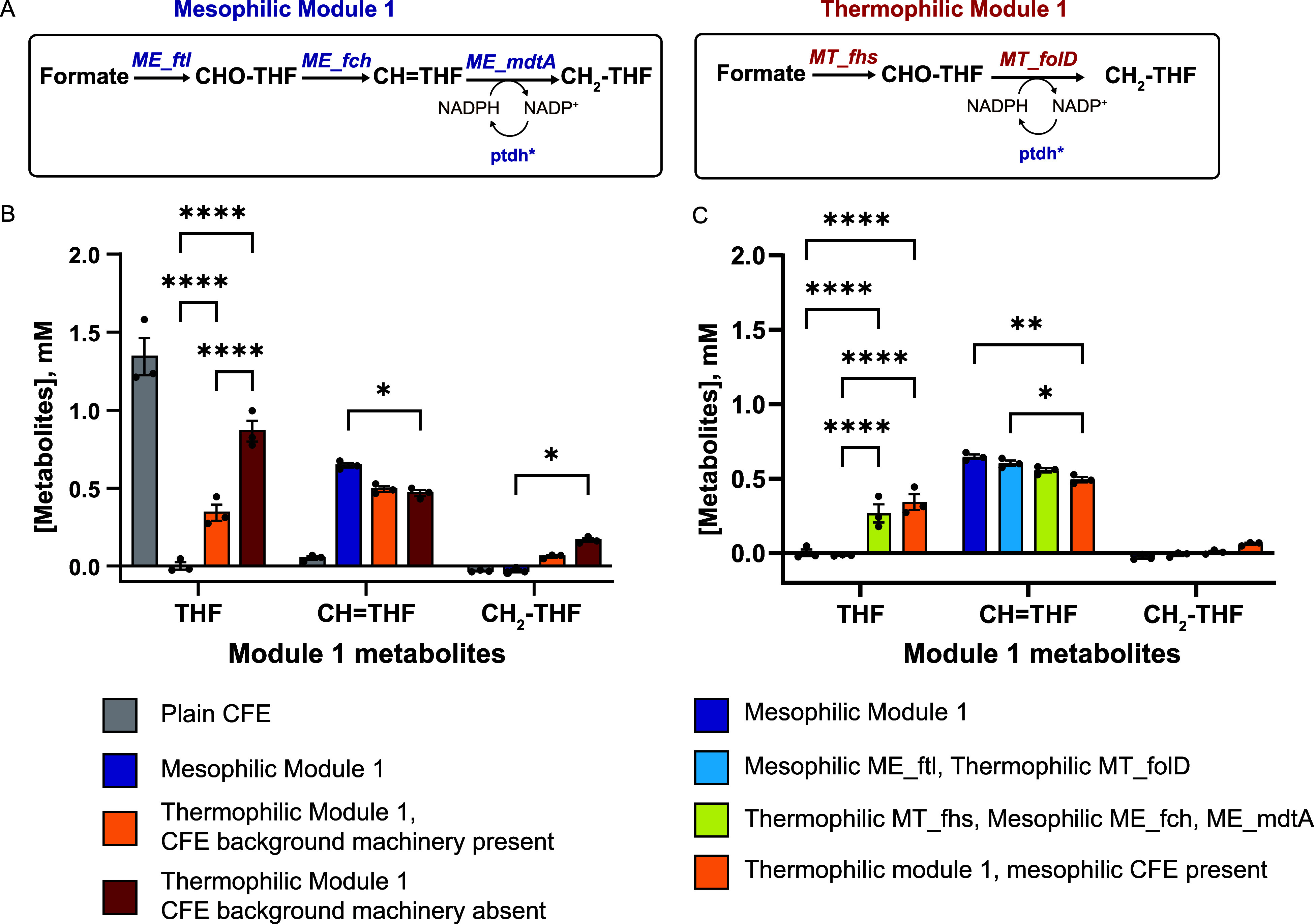
Biosynthetic contribution of thermophilic and mesophilic enzymes in the THF-dependent formate fixation module. (A) Left: Schematic of the mesophilic THF-dependent formate fixation pathway (module 1). Enzyme abbreviations shown in blue: *ME_ftl*, *Methylorubrum extorquens* formate-tetrahydrofolate ligase; *ME_fch*, *M. extorquens* methenyl-THF cyclohydrolase; *ME_mtdA*, *M. extorquens* methylene THF dehydrogenase; ptdh*, phosphite dehydrogenase mutant. Right: Schematic of the thermophilic module 1. Enzyme abbreviations shown in red/blue: *MT_fhs*, *Moorella thermoacetica* formate-tetrahydrofolate ligase; *MT_folD*, *M. thermoacetica* bifunctional protein performing the activities of methenyltetrahydrofolate cyclohydrolase and methylenetetrahydrofolate dehydrogenase; ptdh*, phosphite dehydrogenase mutant. (B) Concentration of module 1 metabolites (THF, CH = THF, and CH_2_–THF) as a function of the module 1 enzyme source (mesophilic, thermophilic) and bioprocess utilized. Of note, heat denaturation of the CFE background machinery was only done in the thermophilic module 1, *CFE* absent samples. Reaction conditions for (B,C**)**: Gene expression: 30 °C, 16 h; biocatalyst dilution: 10×; heat denaturation, 50 °C, 10 min or none; chemical synthesis, 30 °C, 30 min; substrate/cofactor supplementation, 1 mM CH_2_O_2_, 1 mM THF, 1 mM ATP, 2 mM NADPH, 5 mM Na_2_HPO_3_. The bars represent the mean ± standard error of the mean (SEM), *n* = 3, *p < 0.05, ****p < 0.0001. Data were analyzed using a two-way ANOVA followed by a multiple comparisons test via the Tukey method. (C) Concentration of module 1 metabolites (THF, CH = THF, and CH_2_–THF) as a function of the module 1 enzyme source. The CFE machinery is present in all samples (no heat denaturation). The bars represent mean ± SEM, *n* = 3, *p < 0.05, **p < 0.005, ****p < 0.0001. Data were analyzed using a two-way ANOVA followed by a multiple comparisons test via the Tukey method. Metabolite abbreviations: THF, tetrahydrofolate; CH = THF, 5,10-methenyltetrahydrofolate; CH_2_–THF, 5,10-methylenetetrahydrofolate.

As shown in Figure 3B, plain CFE supplemented with 1 mM THF does not synthesize CH = THF or CH_2_–THF. The mesophilic Module 1 converts all THF accumulating CH = THF (0.65 ± 0.02 mM) and has nondetectable levels of CH_2_–THF. As CFE background reactions are known to consume CH_2_–THF,^62^ a C1 donor used in purine, methionine, and thymidylate biosynthesis,^63^ it is possible that ∼0.4 mM of CH_2_–THF were generated and then consumed by CFE-background reactions.

The thermophilic module 1 in the presence of the CFE background machinery converts THF to CH = THF slowly. After 30 min, CH = THF reaches 0.50 ± 0.1 mM with 0.34 ± 0.1 mM THF remaining and limited accumulation of CH_2_–THF (0.01 ± 0.0 mM) is observed. In the absence of the CFE background machinery, the thermophilic module 1 converts THF to CH = THF even more slowly, with 0.87 ± 0.1 mM THF remaining after 30 min, yet accumulates CH_2_–THF to 0.17 ± 0.1 mM. Thus, at the same DNA loading, the mesophilic module 1 converts THF to CH = THF faster than the thermophilic module 1. The removal of the CFE background machinery from thermophilic module 1 enables accumulation of CH_2_–THF, supporting higher glycine/serine synthesis.

Next, we tested module 1 hybrids generated by a mixture of mesophilic and thermophilic enzymes in the presence of the CFE machinery. A module 1 hybrid composed of the mesophilic Ftl and the thermophilic FolD resulted in complete THF conversion, achieving 0.60 ± 0.0 mM CH = THF. A module 1 hybrid composed of the thermophilic Fhs and the mesophilic Fch/MdtA converted THF more slowly, reaching only 0.56 ± 0.0 mM CH = THF and leaving 0.27 ± 0.06 mM unreacted THF. No CH_2_–THF was detectable in either of the module 1 hybrids due to the presence of the CFE background. Taken together, these results show that the presence of the mesophilic CFE machinery limits the accumulation of CH_2_–THF.

### Bioprocess Optimization of the Thermophilic CFE-Based Biocatalyst

To further increase amino acid synthesis yields, we optimized the bioprocess conditions, including (1) heat denaturation temperature, (2) the need to physically separate the denatured mesophilic CFE machinery, (3) chemical synthesis temperature, and (4) pathway gene expression duration (Figure 4).

**Figure 4:**
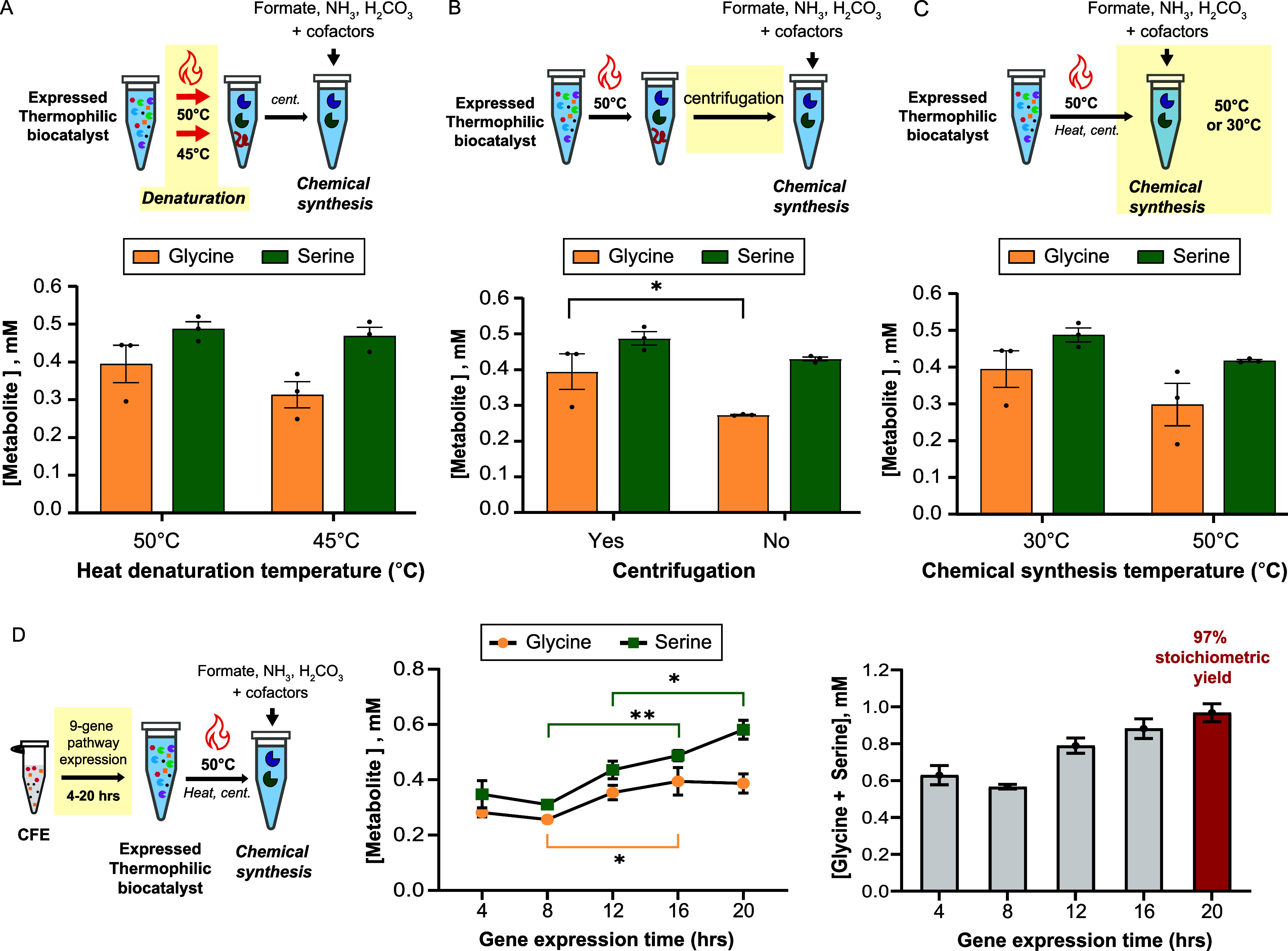
Bioprocess optimization of the CFE-based thermophilic formate-to-serine biocatalyst. Assessment of (A) heat denaturation temperature optimization, (B) the need to physically separate the denatured mesophilic CFE machinery via centrifugation, and (C) chemical synthesis temperature. Reactions conditions for (A–C): Gene expression: 30 °C, 16 h; biocatalyst dilution: 10×; heat denaturation; 50 °C or otherwise noted for 10 min followed by centrifugation or none; chemical synthesis: 30 °C or otherwise noted, 4 h; substrate/cofactor supplementation, 2 mM CH_2_O_2_, 2 mM THF, 1 mM H_2_CO_3_, 1 mM NH_3_, 2 mM ATP, 1 mM NADH, 2 mM NADPH, 5 mM Na_2_HPO_3_. Data shown in (A–C): Bars represent mean ± standard error of the mean (SEM), *n* = 3, *p < 0.05. Data were analyzed using a two-way ANOVA followed by a multiple comparisons test via the Šídák method. (D) Impact of gene expression duration on serine and glycine yields. Middle: Serine and glycine concentrations as a function of gene expression duration. Data points represent mean ± SEM, *n* = 3. Data were analyzed using a two-way ANOVA followed by a multiple comparisons test via the Tukey method. Multiple comparison of glycine concentration at 8 h vs 16 h (*p < 0.05) was statistically significant. Multiple comparisons of serine concentration at 8 h vs 16 h (**p < 0.005) and 12 h vs 20 h (*p < 0.05) were statistically significant. Full representation of all significant comparisons is shown in Figure S3. Right: Combined serine and glycine concentration as a function of gene expression duration. Data points represent mean ± SEM, *n* = 3. Calculations can be found in Table S3.

With regard to the heat denaturation temperature, we initially used 50 °C as *E. coli* enzymes denature above 45 °C,^26^ but *M. thermoacetica* enzymes would not likely be impacted, as *M. thermoacetica* has an optimal growth temperature of 55–60 °C.^54^ As shown in Figure 4A, a heat denaturation step of 50 or 45 °C results in similar serine and glycine concentrations, suggesting no major impact on thermophilic biocatalyst activity. To ensure effective mesophilic CFE machinery denaturation, we used 50 °C in all subsequent experiments.

Next, we determined the impact of physically separating the denatured mesophilic CFE machinery from the thermophilic biocatalyst that remains in solution. As Figure 4B shows, failure to remove the denatured CFE machinery via centrifugation results in a significantly lower concentration of glycine (0.39 mM ± 0.05 mM vs 0.27 mM ± 0.00 mM) but similar concentrations of serine (0.49 mM ± 0.02 mM vs 0.43 mM ± 0.00 mM vs). To maximize product yields, we continued to remove the denatured CFE machinery in subsequent experiments.

Then, we delved into the temperature of the chemical synthesis step. Thus far, we used 30 °C for chemical synthesis to minimize cofactor degradation. High temperatures are known to decrease the stability of several key metabolites and cofactors, with THF degrading rapidly above 37 °C,^46^ THF-based metabolites above 40C,^64–66^ and significant degradation of NAD­(P)H at 50 °C.^67^ However, the *M. thermoacetica* enzymes have an optimal temperature of over 55 °C while the *P. stutzeri* ptdh* mutant has an optimal temperature of 59 °C (Table S1). It is possible that increased enzyme activity may outweigh any cofactor degradation during the chemical synthesis step. As Figure 4C shows, the thermophilic formate-to-serine biocatalyst results in similar serine and glycine yields at 30 and 50 °C. Given that 30 °C is far from the optimal temperature for thermophilic enzyme activity^50,51,68^ and thermal degradation of cofactors is a concern, this suggests the optimal chemical synthesis temperature depends not only on enzyme activity but also on metabolite stability. Therefore, in this thermophilic THF/rGS biocatalyst, any benefit from increased temperature and activity may be outweighed by the thermal instability of the cofactors. Thus, we held the chemical synthesis temperature at 30 °C in subsequent experiments.

Finally, we focused on optimizing the gene expression duration. While a longer gene expression time results—up to a certain point—in more biocatalyst being generated, it also stretches the overall bioprocess run time. Initially, we chose a 16 h gene expression step as cell-free expression of P_T70_-GFP reaches saturation at that time.^13^ Gene expression, however, is affected by the gene sequence and length as well as the number of genes expressed in the one-pot CFE reaction. Thus, we analyzed the impact of varying the pathway gene expression duration on the serine and glycine yields. As shown in Figure 4D, starting at a 12 h gene expression step, the concentration of the intermediate glycine stabilizes at ∼0.39 mM. The concentration of serine, however, steadily increases over time, peaking at 0.58 mM ± 0.03 mM after a 20 h gene expression step. Indeed, after a 20 h gene expression step, the thermophilic formate-to-serine biocatalyst achieves a 0.97 mM combined serine and glycine concentration or 97% of the stoichiometric yield of combined serine and glycine from formate and bicarbonate with a 77.5% formate incorporation (Figure 4D, Tables S2 and S3). As using a 20 h gene expression step was not statistically different than using a 16 h gene expression step (88% of the stoichiometric yield), we used 16 h in all subsequent experiments to reduce the overall process time.

### Impact of Lowering the Cofactor Concentration on Biocatalyst and Product Cost

Key challenges in using enzyme biocatalysts over a microbial biocatalyst for the synthesis of large-scale chemicals are the costs associated with (1) enzyme purification and the (2) exogenous addition of cofactors.^69^ As shown in this work, the CFE-based thermophilic biocatalyst can be separated in bulk from the mesophilic CFE background machinery by rapid and inexpensive heat denaturation. In the CFE-based formate-to-serine biocatalyst, the cost of the cofactors THF ($970/g), NADPH ($428/g), NADH ($60/g), and ATP ($33.6/g) outweighs the cost of the CFE cell lysate ($90/L^16^).^13^ Thus, robust cofactor regeneration is pivotal to reducing the cost of the bioprocess.

Based on previous calculations, a 10-fold diluted CFE-based formate-to-serine biocatalyst costs $1.66/mL^13^, when using equimolar concentrations of substrates and cofactors (2 mM formate, 1 mM NH_3_, 1 mM H_2_CO_3_, 2 mM THF, 2 mM NADPH, 1 mM NADH, 2 mM ATP), i.e., the base case. Figure 5A shows the impact of reducing the initial concentration of THF, NADPH, NADH, and ATP on biocatalyst cost. While reducing the concentration of THF or NADPH by 5-fold reduces the biocatalyst cost by ∼40%, a similar reduction in NADH concentration has a minimal impact on the biocatalyst cost (2% reduction). Combining 5-fold reductions in THF and NADH concentrations lowers the biocatalyst cost drastically, to $0.40/mL. Further reductions in biocatalyst cost require reductions in the concentration of NADH first and ATP last to ultimately achieve $0.09/mL. CFE-based formate-to-serine biocatalysts beyond $0.09/mL will require a reduction in the cost of the CFE lysate. Figure 5B shows the cost of synthesizing serine and glycine using the CFE-based thermophilic formate-to-serine biocatalyst as a function of the reduction in cofactor concentration. In the base case, the cost of serine is $27,299/g while the cost of glycine is $56,833/g. After applying cofactor reductions of THF (25-fold), NADPH (25-fold), NADH (5-fold), and ATP (5-fold), the cost of serine is $1,477/g and the cost of glycine is $3,074/g. Reductions in CFE lysate cost or reuse of the CFE-based catalyst over multiple cycles will be needed to bring down the serine and glycine price to $1–10/g.

**Figure 5:**
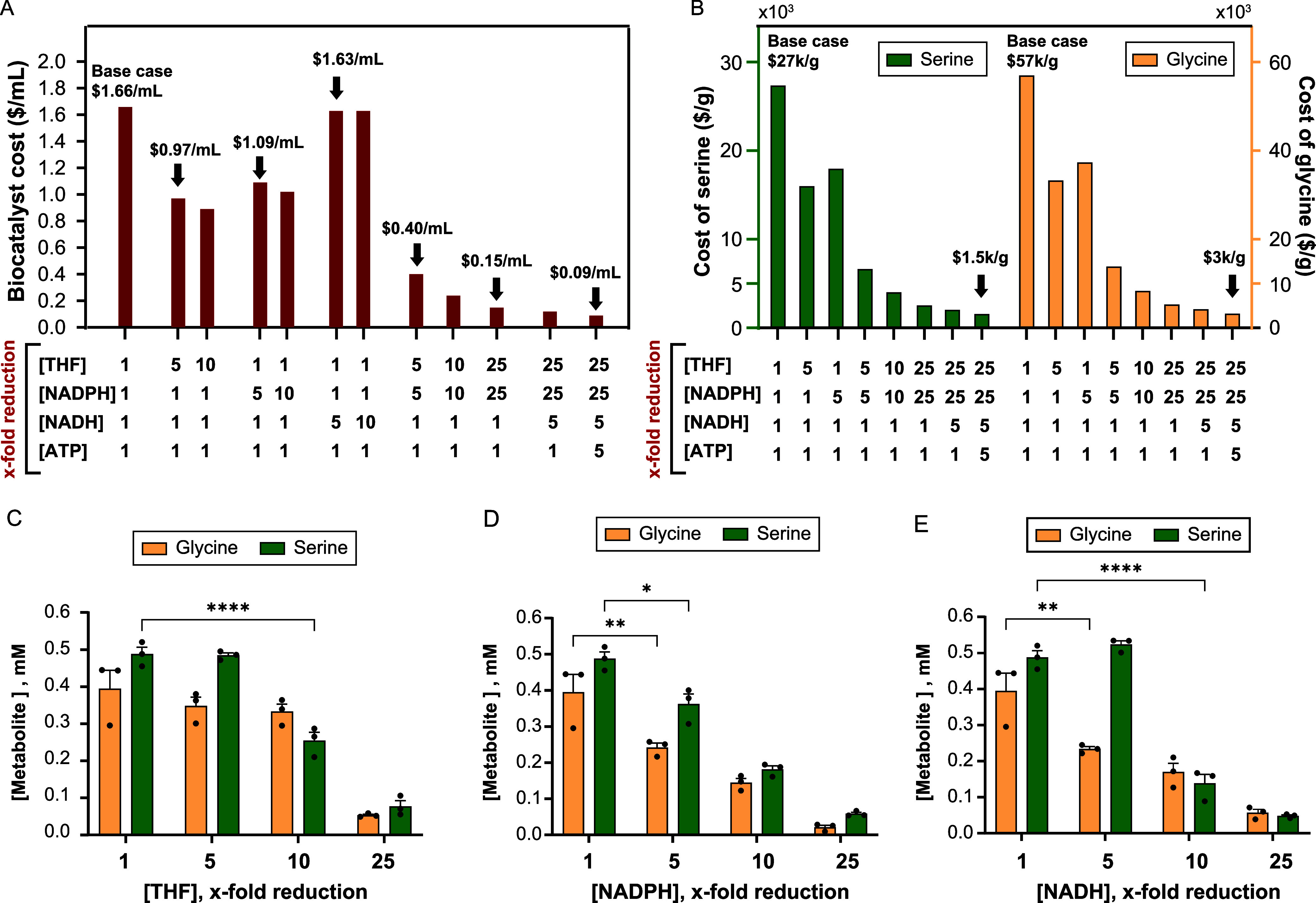
Impact of lowering the concentration of regenerated cofactors on amino acid yields. (A) Biocatalyst cost reduction ($/mL) as a function of cofactor reduction. Calculations are based using a 10-fold diluted CFE-based thermophilic formate-to-serine with an initial cost of $1.66/mL^13^. Base case cofactor concentration (1×-fold [cofactor]): 2 mM THF, 2 mM NADPH, 2 mM NADH, 2 mM ATP. Commercial reagent prices used in cost analysis can be found in Table S11. (B) Cost of serine and glycine from formate produced by the CFE-based thermophilic biocatalyst as a function of cofactor concentration. (C–E) Concentration of serine and glycine from formate as a function of reductions in THF (C), NADPH (D), and NADH (E) concentrations. Reaction conditions: Gene expression: 30 °C, 16 h; biocatalyst dilution, 10×; heat denaturation: 50 °C, 10 min; chemical synthesis: 30 °C, 4 h; substrate/cofactor supplementation, 2 mM CH_2_O_2_, 1 mM H_2_CO_3_, 1 mM NH_3_, 2 mM ATP, 5 mM Na_2_HPO_3_, or the specified cofactor concentration. Bars represent mean ± standard error of the mean (SEM), *n* = 3, *p < 0.05, **p < 0.005, ****p < 0.0001. Data were analyzed using two-way ANOVA followed by a multiple comparisons test via the Tukey method.

### Efficiency of the CFE-Based Thermophilic Formate-to-Serine Biocatalyst at Lower Cofactor Concentrations

To determine how well the CFE-based thermophilic formate-to-serine biocatalyst regenerates THF, NADPH, and NADH, we performed the bioprocess at reduced cofactor concentrations. As shown in Figure 5C, reducing the THF concentration 5-fold (0.4 mM) reduces the biocatalyst cost by 42% ($0.97/mL), while reaching a similar concentration of serine (0.48 ± 0.01 mM) and glycine (0.35 ± 0.02 mM). Reducing the concentration of THF 10-fold (0.2 mM THF) leads to similar glycine concentration but significantly lower serine concentration (0.25 ± 0.02 mM). With respect to NADPH, even a 5-fold reduction in concentration (0.4 mM) significantly reduces the serine (0.36 ± 0.03 mM) and glycine (0.24 ± 0.01 mM) (Figure 5D). We hypothesize that the speed at which NADP^+^ is regenerated to NADPH by ptdh* and the catalytic rate of FolD—the only enzyme that utilizes NADPH as a cofactor—do not match. Lastly, dropping the NADH concentration by 5-fold (0.2 mM) results in similar serine levels but significantly lower glycine concentration at 0.23 ± 0.01 mM (Figure 5E). These data suggest a mismatched activity between NADH recycling by ptdh* and the reductive glycine synthesis module. Finally, we attempted to simultaneously reduce the THF and NADH concentration by 5-fold, but it resulted in a significant reduction of serine and glycine (Figure S4). Taken together, cofactor recycling in the formate-to-serine biocatalyst is complex as THF regeneration is intertwined with NAD­(P)­H regeneration. NADPH regeneration is needed in the THF-formate fixation module that consumes THF, while NADH regeneration is needed in the reductive glycine synthesis module that regenerates THF.

### CFE-Based Thermophilic Formate-to-Serine Pathway as a Fed-Batch Process

The cost of the CFE-based thermophilic biocatalyst process could also be reduced by transitioning from a batch process—where the enzymes are single-use—to a fed-batch process, where the enzymes remain in the reactor and substrates/cofactors are periodically added to the system. For the batch process, we observe plateauing of chemical synthesis after a 4 h chemical synthesis step (Figure 2A). Hypothesizing that the biocatalyst runs out of substrates and cofactors after 4 h rather than the enzymes losing their activity, we used the CFE-based thermophilic biocatalyst in a fed-batch process. Specifically, we supplemented substrates and cofactors at the beginning of the chemical synthesis step (*t* = 0 h) and after 4 h of chemical synthesis (*t* = 4 h) and measured serine and glycine concentration at *t* = 4 h, 8 h, 12 h, and 24 h (Figure 6A).

**Figure 6:**
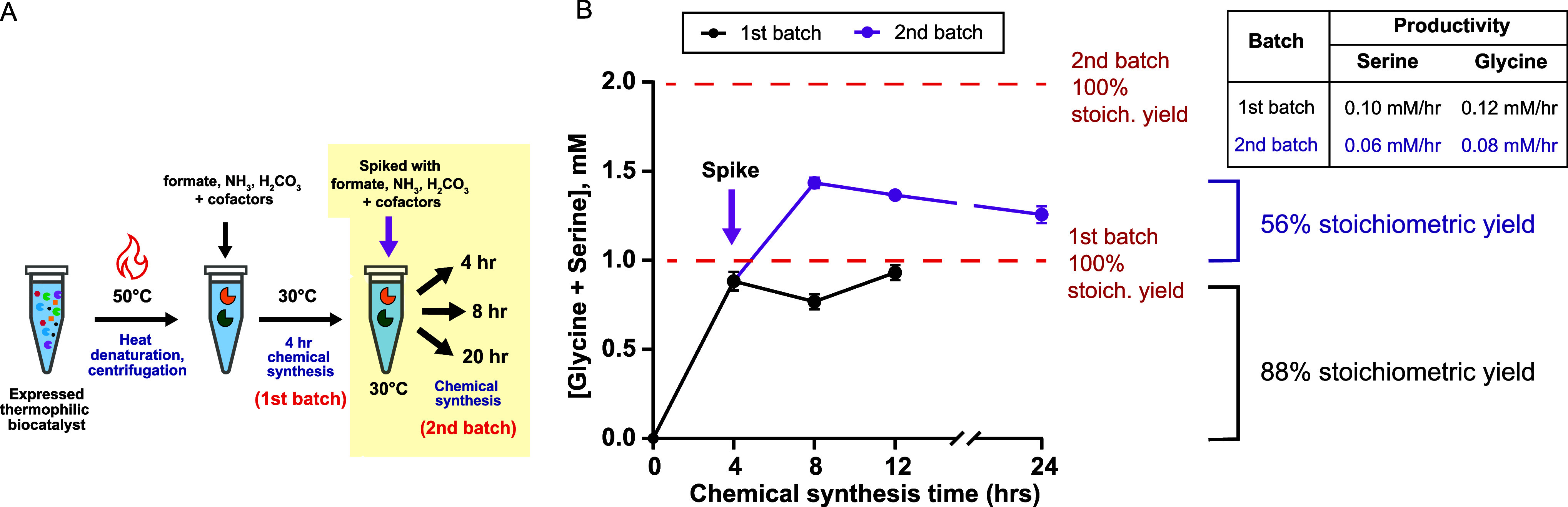
CFE-based thermophilic formate-to-serine biocatalyst fed-batch process: (A) Reaction conditions for fed-batch reactions with protocol changes highlighted in yellow. Complete reaction conditions: Gene expression: 30 °C, 16 h; Biocatalyst dilution, 10×; heat denaturation, 50 °C, 10 min; 2nd batch chemical synthesis, 30 °C, 4–24 h; substrate/cofactor supplementation at first and second batch, 2 mM CH_2_O_2_, 2 mM THF, 1 mM H_2_CO_3_, 1 mM NH_3_, 2 mM ATP, 1 mM NADH, 2 mM NADPH, 5 mM Na_2_HPO_3_. (B) Combined serine and glycine concentration as a function of chemical synthesis time. Chemicals were spiked to initiate a 2nd batch at the 4 h point (purple line and arrow). Black line: reaction performance when chemicals and cofactors are not spiked (data from Figure 2A). Red dotted lines indicate the stoichiometric per-batch yield from the biocatalyst given the initial or spiked substrate/cofactor concentrations. Stoichiometric yield for each batch is indicated next to the graph. Full yield calculations can be found in Table S2. Metabolite production rates from 0–4 h and 4–8 h are indicated in the table on the right. Data points represent mean ± SEM, *n* = 3. Individual serine and glycine synthesis data are shown in Figure S5.

The biocatalyst was generated as previously described and supplemented with equimolar concentrations of substrates and cofactors. After 4 h of chemical synthesis (*t* = 4 h), the biocatalyst generated 0.49 ± 0.02 mM serine (0.12 mM/h ± 0.00 mM/h) and 0.39 ± 0.05 mM glycine (0.10 ± 0.00 mM/h) for an overall 88% of the stoichiometric yield (Figure 6B). The biocatalyst was then spiked with equimolar concentration of substrates and cofactors to run a second batch of the process. During the second 4 h batch (*t* = 8 h), the biocatalyst produced 0.23 mM glycine and 0.33 mM serine or 56% of the stoichiometric yield with a productivity of 0.08 ± 0.00 mM/h serine and 0.06 ± 0.01 mM/h glycine (Figure 6B, Tables S2 and S3). Therefore, the biocatalyst was still active during the second fed batch—however, its biosynthetic productivity dropped by ∼40% from batch to batch. Beyond 8 h (*t* = 12 h and *t* = 24 h), the combined concentration of serine and glycine plateaus (Figure 6B). These results show that the CFE-based thermophilic formate-to-serine biocatalyst can be used in a fed-batch process with a ∼25% decrease in product yield from batch to batch. In the future, introduction of a third and a fourth batch will help determine the limits of the CFE-based biocatalyst.

## Discussion

In this work, we used a mesophilic *E. coli* lysate-based CFE to generate a 9-gene thermophilic formate-to-serine biocatalyst in a one-pot reaction for the synthesis of serine and glycine from formate, bicarbonate, and ammonia. The thermophilic nature of the biocatalyst enabled its rapid and cost-effective purification away from the mesophilic CFE machinery, which siphons the amino acid products, in-pathway intermediates, and cofactors if not removed. The thermophilic biocatalyst was built using enzymes from the moderate thermophile *M. thermoacetica*, which allowed the chemical synthesis to be run at mesophilic temperatures, which limited thermal cofactor degradation. The thermophilic biocatalyst performance did not improve when running the chemical synthesis at moderately thermophilic temperatures, supporting the hypothesis that optimal enzyme activity and thermal cofactor degradation need to be balanced when using thermophilic enzymes. Ultimately, the CFE-based thermophilic biocatalyst achieves a 97% of the stoichiometric yield of combined serine and glycine from one-carbon feedstocks using a 20 h gene expression step and a 4 h chemical synthesis. Finally, with an eye toward process scalability, we demonstrate the potential of using a CFE-based biocatalyst in a fed-batch process over 8 h of performance. To the best of our knowledge, this is the first instance of expressing enzymes from a thermophilic organism for chemical synthesis and utilizing those enzymes at a mesophilic temperature in an *E. coli*-based CFE system.

The commercial feasibility of CFE-based biocatalysts will depend on lowering the cost of the cell lysate ($90/L^16^) and the cofactors. In this work, we lowered the bioprocess cost by reducing the cofactor loading. With a cost of $1.66/mL^13^, the current CFE-based thermophilic biocatalyst produces serine and glycine at a cost of $27,299/g and $56,833/g, respectively. Reducing the concentration of the most expensive cofactor, THF, by 5-fold did not significantly reduce the yield of glycine or serine from formate while allowing a 42% reduction in bioprocess cost ($1.66/mL to $0.97/mL). The biocatalyst does not regenerate ATP—the least expensive cofactor—which does not significantly affect the biocatalyst cost. Nevertheless, in the future, ATP regeneration could be implemented with a thermophilic polyphosphate kinase, as has been demonstrated in multiple systems.^28,70,71^ Reductions in cofactor concentration could bring the cost of the biocatalyst down to $0.09/mL, reducing the cost of serine and glycine to $1,477/g and $3,074/g, respectively. Further reductions in biocatalyst cost and ultimately product cost will come from reducing the cost of the cell lysate or the ability to reuse the biocatalyst in a multi-fed-batch or continuous process.

Toward developing a continuous process, we assess the ability of the biocatalyst in a fed-batch system and observe limitations with the CFE-based biocatalyst activity lifetime. In this work, the thermophilic biocatalyst displayed sustained activity over an 8 h period with 2 batches of reagents (substrates and cofactors) with a 24% reduction in the product yield from batch to batch. In the future, the bioprocess could be extended beyond two fed batches to a multibatch or continuous process to push the limits of the biocatalyst lifetime. Furthermore, the biocatalyst could be spiked only with substrates and not with cofactors to make full use of the cofactor regeneration systems.

Future improvements to the thermophilic biocatalyst process will require a closer examination of pathway kinetics. For example, the current biocatalyst shows a biosynthetic efficiency imbalance between module 2 and module 3, both of which use CH_2_–THF, as glycine is accumulated in the system. Specifically, module 2 efficiently combines CH_2_–THF and H_2_CO_3_ to generate glycine, but module 3 is not able to combine all of the glycine with CH_2_–THF to generate serine. This indicates a pathway bottleneck in either module 3 or module 1 that generates CH_2_–THF from formate. Future investigation of pathway intermediates will determine the best places for optimization. Increasing cofactor recycling or using higher-activity enzyme homologues could improve individual modules. Balancing the biosynthetic efficiency of glycine conversion could be achieved by altering the module 2/module 3 gene ratios or timing the degradation of module 2 enzymes to facilitate the complete conversion of formate into serine. In this work, we investigated the activities of the module 1 enzymes while future studies should examine the Shmt enzyme of module 3—the most thermodynamically unfavorable step^13^—to improve serine production. Extending the pathway to other products is also an exciting possibility, as serine is just one step from pyruvate, a gateway to multiple bioproducts. This could provide a sink for serine to pull metabolites toward a single product. Whole-pathway insight could ultimately be gained through data-driven machine learning or kinetic models in which heat denaturation provides a significant advantage. Without interference from the background metabolism, the pathway can be more accurately investigated, resulting in more powerful models and predictions in the chemical yield.

## Materials and Methods

### Materials

All materials, including chemicals, solvents, kits, plasmids, primers, and gene sequences, can be found in the Supporting Information Tables S4–S10.

### Bioinformatics Analysis

BRENDA^49^ and Uniprot^72^ were used to identify thermophilic enzymes with known experimental catalytic and thermostability parameters. Module 1 thermophilic enzymes were chosen based on their optimal kinetic and thermostability parameters at moderately thermophilic temperatures (50–70 °C). There were no experimental parameters for thermophilic glycine cleavage pathway (Gcv) enzymes or serine hydroxymethyltransferase (Shmt). Gene sequences for *gcv* and *shmt* were identified based on protein homology and automated gene predictions from the BioCyc database.^58^

### Thermophilic Formate-to-Serine Pathway Construction

*M. thermoacetica* *fhs*, *foldD, gcvP, gcvH, gcvT, gcvL, lipM,* and *shmt* were codon optimized for *E. coli* and commercially synthesized. Genes were cloned under control of the P_T70_ promoter in plasmid pTX/TL-P70a-deGFP between *NdeI*/*XhoI* using Gibson assembly. Clones were confirmed by whole plasmid DNA sequencing. The plasmid carrying ptdh* (p70a-*P. stutzeri*_ptdh*) has been previously disclosed.^13^ To generate the linear DNA for use in the CFE, the nine genes (genes *fhs*, *foldD*, *gcvP*, *gcvH*, *gcvT*, *gcvL*, *lipM*, *shmt*, and ptdh*) were amplified from their respective vectors using RW9/RW10 that bound ∼100bp upstream from the promoter and downstream from the terminator to protect the sequence from exonuclease degradation.

### Synthesis of Serine and Glycine from Formate, Bicarbonate, and Ammonia

Reactions were set up in a 96-well plate using a Labcyte Echo 525. The reactions contained 100 μM PLP, 100 μM α-lipoic acid (dissolved in water), 3 nM P_T70_-*M. thermoacetica*_fhs, 3 nM P_T70_-*M. thermoacetica*_folD, 192 nM P_T70_-*M. thermoacetica*_gcvH, 2 nM P_T70_-*M. thermoacetica*_gcvL, 1 nM P_T70_-*M. thermoacetica*_gcvP, 4 nM P_T70_-*M. thermoacetica*_gcvT, 2 nM P_T70_-*M. thermoacetica*_lipM, 3 nM P_T70_-*M. thermoacetica*_shmt, and 3 nM P_T70_-*P. stutzeri*_ptdh*. By hand, the transcription/translation mixture (TX/TL, 75% vol) and water were added to reach 5 μL. Gene expression step: 4–20 h at 30 °C and shaken at 1.8*g*. Biocatalyst dilution step: 10× dilution was obtained by moving the biocatalyst to a PCR tube and adding 0.1 M Tris HCL at pH 8 to reach 50 μL. Heat denaturation step: The biocatalyst was overlaid with argon, incubated at 50 °C for 10 min, and centrifuged for 10 min to pellet the precipitated *E. coli* CFE background machinery. Chemical synthesis step: The biocatalyst supernatant was moved to a new PCR tube and supplemented with substrate, cofactors, and buffer to reach 50 μL. Final concentrations: 20 mM DTT, 100 μM α-lipoic acid (dissolved in DMSO), 2 mM THF, 2 mM formate, 2 mM NADPH, 2 mM ATP, 1 mM NH_3_, 1 mM NaHCO_3_, 1 mM NADH, and 5 mM Na_2_HPO_3_. Reactions with 5×, 10×, and 25× reduced THF concentration contained 0.4 mM, 0.2 mM, and 0.08 mM THF, respectively. Reactions with 5×, 10×, and 25× reduced NADPH concentration contained 0.4 mM, 0.2 mM, and 0.08 mM NADPH, respectively. Reactions with 5×, 10×, and 25× reduced NADH concentration contained 0.2 mM, 0.1 mM, and 0.04 mM NADH, respectively. The reactions were overlaid with argon and sealed to establish semianaerobic conditions. Chemical synthesis took place for 2–12 h at 30 °C shaken at 1.8*g*.

### NADPH Consumption by Plain CFE with and without Heat Denaturation

Reactions were set up in a 96-well plate containing a TX/TL mixture (75% vol) and water to reach 5 μL. Reactions were incubated for 16 h at 30 °C and shaken at 1.8*g*. Plain CFE dilution step: 10× dilution was obtained by moving the CFE reaction mixture to a PCR tube and adding 0.1 M pH 8 Tris HCL to reach 50 μL. Heat denaturation step: Reactions that were heat denatured were incubated at 50 °C for 10 min and centrifuged for 10 min to pellet the precipitated *E. coli* CFE machinery. Heat-denatured supernatants were then moved to a new PCR tube and all reactions were supplemented with 1 mM NADPH. Zero-hour reactions were measured immediately while all other reactions were incubated for 2–12 h at 30 °C shaken at 1.8*g*.

### Module 1 Reactions: Synthesis of CH_2_–THF from Formate

Reactions were set up in a 96-well plate using a Labcyte Echo 525. The reactions contained combinations of 3 nM P_T70_-*M. thermoacetica*_fhs, 3 nM P_T70_-*M. thermoacetica*_folD, P_T70_-*E. coli* *_folD*, P_T70_-*M. extorquens*_ftl, P_T70_-*M. extorquens*_fch, and P_T70_-*M. extorquens*_mtdA. All reactions contained 3 nM P_T70_-*P. stutzeri*_ptdh*. By hand, TX/TL (75% vol) and water were added to reach 5 μL. Gene expression step: 16 h at 30 °C shaken at 1.8*g*. Biocatalyst dilution step: 10× dilution was obtained by moving the biocatalyst to a PCR tube and adding 0.1 M pH 8 Tris HCL to reach 50 μL. Heat denaturation step: For heat-denatured samples, the biocatalyst was overlaid with argon, incubated at 50 °C for 10 min, and centrifuged for 10 min to pellet the precipitated *E. coli* CFE machinery. Chemical synthesis step: For heat-denatured samples, the biocatalyst supernatant was moved to a new PCR tube. All reactions were supplemented with substrate, cofactors, and buffer to reach 50 μL. Final concentrations: 1 mM THF, 1 mM formate, 1 mM NADPH, 1 mM ATP, and 5 mM Na_2_HPO_3_. The reactions were overlaid with argon and sealed to establish semianaerobic conditions. Chemical synthesis took place for 30 min at 30 °C and shaken at 1.8*g*.

### Fed-Batch Synthesis of Serine and Glycine from Formate, Bicarbonate, and Ammonia

Reactions were set up and incubated by following the same protocol as the batch reactions. After the first 4 h chemical synthesis step, tubes were unsealed and all chemicals (20 mM DTT, 100 μM α-lipoic acid (dissolved in DMSO), 2 mM THF, 2 mM formate, 2 mM NADPH, 2 mM ATP, 1 mM NH_3_, 1 mM NaHCO_3_, 1 mM NADH, and 5 mM Na_2_HPO_3_) were spiked into the reaction in an additional 5 μL of volume to reach the same initial concentrations (10% volume increase assumed to be negligible). The reactions were then overlaid with argon and resealed. Chemical synthesis was resumed for an additional 4–20 h at 30 °C and shaken at 1.8*g*.

### Amino Acid Derivatization

For liquid chromatography/mass spectrometry (LC/MS) detection and quantification of serine and glycine, the amino acids were derivatized to their Fmoc-protected versions using 9-fluorenylmethoxycarbonyl chloride (Fmoc-Cl)^73^ and yield calculations were done assuming 100% derivatization efficiency in both the standard curves and experimental data (Figure S6). To the 10× diluted biocatalyst (50 μL) was added 50 μL of 5% acetic acid in methanol containing 2 mM Boc-serine (internal standard) for a final concentration of 1 mM Boc-serine in the reaction. The denatured reaction was centrifuged for 10 min, and 25 μL of the supernatant was moved to a new tube. The pH was adjusted to 8.3 using 50 μL of saturated NaHCO_3_ and then 100 μL of 3 mM Fmoc-Cl dissolved in acetone was added. The reactions were performed at room temperature for 10 min. The Fmoc-derivatized amino acids were extracted using 500 μL of ethyl acetate (3×), dried under a vacuum, and resuspended in 200 μL of methanol. Derivatized amino acids were spun at 21,000*g* for 15 min, and then 100 μL of the supernatant was removed for analysis.

### Metabolite Quantification

The Fmoc-derivatized amino acids were quantified using an Agilent 1260 Infinity II HPLC system equipped with an Agilent Q-TOF 6530 detector using a Poroshell 120 SB-C_18_ 3.0 × 50 × 2.7 μm column. LC conditions: Solvent A—water with 0.1% formic acid, solvent B—acetonitrile with 0.1% formic acid. Gradient: 0 min, 5% B; 3 min, 5% B; 15 min, 100% B; 18 min, 100% B; 20 min, 5% B; 22 min, 5% B. MS acquisition: Extracted ion chromatograms in positive ion mode were used to detect and quantify Fmoc serine (*m*/*z* 328.11) and Fmoc glycine (*m*/*z* 298.11). Fmoc-derivatized commercial serine and glycine were used to determine retention times and generate standard curves for chemical quantification.

All other metabolites were detected and quantified via LC/MS without derivatization using an Agilent 1260 Infinity II HPLC system equipped with an Agilent LC/MSD iQ Single Quadrapole MS and an electrospray ion source using a Poroshell 120 EC-C_18_ 2.1 × 50 × 2.7 μm column. The proteins in the 10× diluted biocatalyst were denatured by adding 50 μL of 5% acetic acid in methanol spiked with 0.1 mM catechol (internal standard). The denatured reactions were centrifuged at 3,600*g* for 10 min, and the supernatant was directly analyzed. Column temperature was kept constant at 28 °C. LC conditions: Solvent A—water with 3% methanol, 10 mM tributylamine, and 15 mM acetic acid, solvent B—methanol. Gradient: 0 min, 0% B; 1 min, 0% B; 2 min, 65% B; 4.5 min, 77.5% B; 5 min, 95% B; 6 min, 0% B; and 8.5 min, 0% B. MS acquisition: negative ion scan was used to extract ion chromatograms and quantify THF (*m*/*z* 444), CH = THF (*m*/*z* 454), CH_2_–THF (*m*/*z* 456), NADP+ (*m*/*z* 742), and NADPH (*m*/*z* 744). Commercial THF, CH = THF, CH_2_–THF, NADPH, and NADP^+^ were used to determine retention times and generate standard curves for chemical quantification.

## Supplementary Material



## Data Availability

All the data generated or analyzed during this study is included in the published article and its Supporting Information files.

## Notes

Properties of the enzymes in the thermophilic formate-to-serine biocatalyst; stoichiometric yield calculations; formate incorporation calculations; table of reagents; table of solvents; table of kits; table of plasmids; table of primers; table of promoter and terminator; sequences of genes evaluated; table of commercial reagent prices used in cost analysis; images of plain CFE and the CFE-based thermophilic module 1 biocatalyst during different steps of the bioprocess workflow; stability of CH_2_–THF at 30 °C over time; extended comparisons between groups shown in Figure 4D; concentration of glycine/serine from formate as a function of simultaneous reduction in THF and NADH concentrations; individual serine and glycine concentrations shown in Figure 6B; efficiency of Fmoc derivatization of serine and glycine; LC/MS traces of commercial Fmoc serine and Fmoc glycine in plain cell-free expression (CFE); standard curves of Fmoc serine and Fmoc glycine using derivatized Fmoc serine and glycine under the bioprocess workflow conditions used to quantify serine and glycine concentrations in this study; LC/MS traces of commercial tetrahydrofolate (THF), 5,10-methenyltetrahydrofolate (CH = THF), 5,10-methylenetetrahydrofolate (CH_2_–THF), NADPH, and NADP+ in plain cell-free expression (CFE); and standard curves using commercial tetrahydrofolate (THF), 5,10-methenyltetrahydrofolate (CH = THF), 5,10-methylenetetrahydrofolate (CH_2_–THF), NADPH, and NADP^+^ (PDF)
