# Supplementary material for: Cell-Free-Based Thermophilic Biocatalyst for the Synthesis of Amino Acids from One-Carbon Feedstocks
Source: ACS Synth Biol. 2025 Oct 18;14(11):4424–38. doi: 10.1021/acssynbio.5c00352 (PMC12645577; doi:10.1021/acssynbio.5c00352)
Supplement: Supplementary file 1 [file sb5c00352_si_001.pdf]

Cell-free-based thermophilic biocatalyst for the synthesis of amino acids from one-carbon feedstocks

Ray Westenberg[1,2], Shaafique Chowdhury[1], Ryan Cardiff[3], Kimberly Wennerholm[4], Alexander S. Beliaev[5,6], James M. Carothers[3,7*], Pamela Peralta-Yahya[1,2,3,8*]

[1] School of Chemical & Biomolecular Engineering, Georgia Institute of Technology, Atlanta, GA 30332

[2] Bioengineering Program, Georgia Institute of Technology, Atlanta, GA 30332

[3] Molecular Engineering & Sciences Institute and Center for Synthetic Biology, University of Washington, Seattle, WA 98195, USA

[4] School of Chemistry and Biochemistry, Georgia Institute of Technology, Atlanta, GA 30332

[5] Environmental Molecular Sciences Division, Pacific Northwest National Laboratory, Richland, Washington 99354, United States

[6] Centre for Agriculture and the Bioeconomy, School of Biological and Environmental Sciences, Queensland University of Technology, Gardens Point Campus, P.O. Box 2434, Brisbane 4001, Queensland, Australia

[7] Department of Chemical Engineering, University of Washington, Seattle, WA 98195, USA

[8] Lead contact

* Correspondence to:

E-mail: jcaroth@uw.edu, pperalta-yahya@chemistry.gatech.edu

Supplementary Information

Table of Contents

	Page
**Table S1.** Properties of the enzymes in the thermophilic formate-to-serine biocatalyst	SI3
**Table S2:** Stoichiometric yield calculations	SI4
**Table S3:** Formate incorporation calculations	SI5
**Table S4.** Table of Reagents	SI6
**Table S5.** Table of Solvents	SI6
**Table S6.** Table of Kits	SI7
**Table S7.** Table of Plasmids	SI7
**Table S8.** Table of Primers	SI7
**Table S9.** Table of Promoter and terminator	SI8
**Table S10.** Sequences of genes evaluated	SI8
**Table S11.** Table of commercial reagent prices used in cost analysis	SI11
**Figure S1:** Images of Plain CFE and the CFE-based thermophilic Module 1 biocatalyst during different steps of the bioprocess workflow.	SI12
**Figure S2**: Stability of CH_2-THF at 30°C over time.	SI13
**Figure S3:** Extended comparisons between groups shown in Figure 4D.	SI14
**Figure S4:** Concentration of glycine/serine from formate as a function of simultaneous reduction in THF and NADH concentrations.	SI15
**Figure S5:** Individual glycine and serine concentrations shown in Figure 6B.	SI16
**Figure S6**: Efficiency of Fmoc derivatization of serine and glycine.	SI17
**Figure S7**: LC/MS traces of commercial Fmoc-Serine and Fmoc-glycine in plain cell-free expression (CFE).	SI18
**Figure S8.** Standard curves of Fmoc-Serine and Fmoc-Glycine using derivatized Fmoc serine and glycine under the bioprocess workflow conditions used to quantify glycine and serine concentrations in this study	SI19
**Figure S9**: LC/MS traces of commercial tetrahydrofolate (THF), 5,10-methenyltetrahydrofolate (CH=THF), 5,10 methyleneltetrahydrofolate (CH_2-THF), NADPH, and NADP+ in plain cell-free expression (CFE).	SI20
**Figure S10**: Standard curves using commercial tetrahydrofolate (THF), 5,10- methenyltetrahydrofolate (CH=THF) and 5,10 methyleneltetrahydrofolate (CH_2-THF), NADPH and NADP+.	SI21
**References**	SI22

Table S1. Properties of the enzymes in the thermophilic formate-to-serine biocatalyst

Pathway	Enzyme		Source	T_{opt} (°C)	Thermo stability	Specific activity (Units, U: µmol min^{-1} mg^{-1})	Protein identity to mesophilic homolog (%)[13]	Protein coverage to mesophilic homolog (%)[13]
THF-dependent formate fixation	fhs	6.3.4.3	*Moorella thermoacetica*	55-60[46]	Tm = 69°C[46]	780 (at 50°C)[46]	60.68	99
	folD	1.5.1.5 3.5.4.9		64[47]	Stable at 50°C, retains 80% activity at 61°C[47]	720 (at 60°C), 200 (at 30°C)[48]	35.42	66
Reductive glycine synthesis	gcvT	1.4.1.2 7		--	--	--	40.21	99
	gcvH			--	--	--	54.69	97
	gcvP			--	--	--	35.25	100
	gcvL			--	--	--	39.47	98
	lipM	2.3.1.1 81		--	--	--	24.18	97
Serine synthesis	shmt	2.1.2.1		--	--	--	58.64	99
NAD(P)H recycling	ptdh*	-	*Pseudomon as stutzeri*	59[55 a]	T_m = 59 °C, $t_{1/2}$= 6 days at 45°C[55 a]	12.2 (at 30°C)[56 b]	N/A	N/A

[a] 12X mutant

[b] Native enzyme

Table S2. Stoichiometric yield calculations

		CH_2O_2 (mM)	H_2CO_3 (mM)	Max. stoich. serine + glycine (mM)	Serine (mM)	Glycine (mM)	Stoichiometric yield (%)
Figure 1D	CFE plain	2	1	1	0.06	0.05	11
	Mesophilic biocatalyst	2	1	1	0.19	0.11	30
	Thermophilic biocatalyst - 16 hrs gene express	2	1	1	0.49	0.39	88
Figure 4D	Thermophilic biocatalyst - 20 hrs gene express.	2	1	1	0.58	0.39	97
Figure 6B	Thermophilic biocatalyst - 1st batch	2	1	1	0.49	0.39	88
	Thermophilic Biocatalyst- 2nd batch	2	1	1	0.33	0.23	56

Table S3. Formate incorporation calculations

			Serine			Glycine				
		CH_2O_2 (mM)	**Serine actual (mM)**	**CH_2O_2 incorp. (mM)**	**Remaining CH_2O_2**	**Glycine actual (mM)**	**CH_2O_2 incorp. (mM)**	**Remaining CH_2O_2**	**CH_2O_2 incorp into Ser & Gly (mM)**	**CH_2O_2 incorp (%)**
**Figure 1D**	CFE plain	2	0.06	0.12	1.88	0.05	0.05	1.83	0.17	**8.5**
	Mesophilic biocatalyst	2	0.19	0.38	1.62	0.11	0.11	1.51	0.49	**24.5**
	Thermophilic biocatalyst	2	0.49	0.98	1.02	0.39	0.39	0.63	1.37	**68.5**
**Figure 4D**	Thermophilic biocatalyst - 20 hours gene expression	2	0.58	1.16	0.84	0.39	0.39	0.45	1.55	**77.5**
**Figure 6B**	Thermophilic biocatalyst - 1st batch	2	0.49	0.98	1.02	0.39	0.39	0.63	1.37	**68.5**
	Thermophilic biocatalyst - 2nd batch	2	0.33	0.66	1.34	0.23	0.23	1.11	0.89	**44.5**

Table S4. Table of reagents.

Reagents	Vendor	Catalog #
1,4-dithiothreitol (DTT)	Millipore Sigma	3483-12-3
(6R,S)-5,10-Methenyl-5,6,7,8-tetrahydrofolic Acid (CH=THF)	Cayman Chemicals	31333
25% ammonia in water	Millipore Sigma	1.05422
α-lipoic acid	Millipore Sigma	1368301
ATP	Millipore Sigma	A6419
Catechol	Millipore Sigma	PHL823720
Fmoc Chloride	Oakwood Chemical	022072
Folitixorin (CH_2-THF)	Cayman Chemicals	33967
Formic acid	Fischer scientific	A117-50
Glycine	Millipore Sigma	G7126
NADH	Cayman Chemicals	16078
NADPH	Cayman Chemicals	9000743
Na_2HPO_3	Millipore Sigma	04283
Pyridoxal-5-phosphate	TCI chemicals	C0377
Serine	Millipore Sigma	S4500
Sodium bicarbonate	Millipore Sigma	S5761
Tetrahydrofolate	Cayman Chemicals	18263

Table S5. Table of solvents

Reagents	Vendor	Catalog #
Acetic acid	EMD Millipore	101830
Methanol	Fischer Scientific	A452-4
Tributylamine	Sigma	90780
Ethyl Acetate	Sigma	319902
Acetone	Fischer Scientific	326801000

Table S6. Table of kits

Reagents	Vendor	Catalog #
myTXTL Linear DNA master mix	Arbor Biosciences	508096

Table S7. Table of plasmids

Strain number	Plasmid name	Description	Source
PPY2510	pTXTL-P70a-deGFP		Arbor Biosciences #502056
PPY2541	pRW25	p70a-*P.stutzeri*_17X-PTDH	[1]
PPY2610	pSC38	p70a-*M.extorquens*_ftl	[1]
PPY2536	pRW20	p70a-*M.extorquens*_fch	[1]
PPY2611	pSC39	p70a-*M.extorquens*_mtdA	[1]
PPY2530	pRW14	p70a-*M.thermoacetica*_fhs	This Study
PPY2543	pRW27	p70a-*M.thermoacetica*_folD	This Study
PPY2531	pRW15	p70a-*M.thermoacetica*_gcvT	This Study
PPY2532	pRW16	p70a-*M.thermoacetica*_gcvH	This Study
PPY2533	pRW17	p70a-*M.thermoacetica*_gcvP	This Study
PPY2534	pRW18	p70a-*M.thermoacetica*_gcvL	This Study
PPY2508	pRW8	p70a-*M.thermoacetica*_lipM	This Study
PPY2535	pRW19	p70a-*M.thermoacetica*_shmt	This Study

Table S8. Table of primers

Primer Name	Sequence
RW9	GACTATCGCACCATCAGC
RW10	CTGTCCTACGAGTTGCATG

Table S9. Table of Promoter and Terminator

Promoter	Sequence
P_{T70}	TGAGCTAACACCGTGCGTGTTGACAATTTTACCTCTGGCGGTGATAATGGTTGCA**<u>GCTAGC</u>**
T_{500}	**<u>CTCGAG</u>**CAAAGCCCGCCGAAAGGCGGGCAAAGCCCGCCGAAAGGCGGGCTTTTCTGT

Nhe, NcoI, XhoI sites **<u>bold and underlined</u>**

Table S10. Sequences of genes evaluated

Source	Gene	Enzyme	Notes	Sequences used
Moorella thermoacetica	*fhs*	formate-THF ligase	Q2RM91 (*E.coli* codon optimized)	atgtctaaagtaccctctgatattgagattgcgcaagctgccaaaatgaaaccggtcatggaa<br>ctggcccgtggactgggcattcaagaggacgaagtagaactttacggaaagtataaggca<br>aaaatttctctcgatgtctatcgtcgcctcaaagacaaacctgacgggaaactaattctggtaa<br>ctgccattactcctactccggccggcgaaggtaagactactacttctgtcggtctcactgatgcc<br>ctggctcgcctggggaaaagggtgatggtctgtctgcgggagccctctctgggaccctcttttg<br>gtattaaaggcggtgccgccggcggtggttatgcccaggtagtaccaatggaagatattaatc<br>tgcactttactggcgatattcacgccgtcacttacgcccacaatctgctggcggcaatggtgga<br>taatcacctgcaacagggtaatgtcctgaatattgatccccgtacaataacttggcgtcgtgta<br>atagaccttaatgaccgtgctctgcgtaatattgtcattggcctgggtggcaaagccaacggc<br>gtaccacgggagactgggtttgacatttctgttgcctctgaagtaatggcctgtctgtgtctggca<br>tcagacctcatggatctcaaagaacgcttttctcgcattgttgttggttatacttacgacggcaaa<br>ccggtcactgccggcgatctggaggcccagggttctatggctcttctcatgaaagatgcaata<br>aagccgaacctggtacaaactctggaaaatactcctgccttcatccacggtggtccctttgcc<br>aatattgcccacggttgtaattctattatcgcgaccaaaactgccctgaaacttgccgactacgt<br>cgttaccgaagccggttttggtgccgacctgggcgcagagaaattctatgatgttaaatgtcgtt<br>acgccggctttaaacccgacgccactgtcattgtggctactgtccgcgccctcaaaatgcacg<br>gcggcgtacccaaatctgacctggccactgaaaatctggaagccctgcgggaaggctttgc<br>caatctggagaaacacattgaaaacattggcaaatttggcgtaccggcagtcgtggccatta<br>atgcctttcccactgatactgaggccgagctaaatctcctctacgaactctgcgccaaagctg<br>gggccgaagttgccctctctgaagtctgggctaaaggcggcgaaggcggtctggaacttgc<br>ccggaaagtgttgcagactctggagtctaggccatctaattttcacgttctgtacaatctggacc<br>tgtctatcaaggataaaattgccaaaattgccactgagatttacggggcagacggcgtcaatt<br>atactgccgaagccgacaaagctattcagcgttatgaatctctgggctatggcaatctgccgg<br>tggtcatggccaaaactcaatattcattttctgatgacatgactaaactggggcgtccgcgtaat<br>tttactattactgtgcgcgaggtgcgcctctctgccggaggcaggcttattgtccccattactggc<br>gccattatgactatgccagggctgcccaaacgcccagccgcgtgtaatattgacattgatgcc<br>gacggtgtgatcactggtcttttctag

	folD	Bifunctional methenyl-THF cyclohydrolase and methylene THF dehydrogase	Q2RIB4 (*E.coli* codon optimized)	atgccagctcaaattctcgacgggaagaaaattgctgccgaagtacgtgcagaggtaaaag<br>aggaagtttcccggttaaaagcagaaggtattaatccaggtctggccgtagtcctggtgggcg<br>aagacccggcttcccaagtgtatgttagaaataaacaccgcgcctgtgaggaggttggtatct<br>attccgaggttcaccgcctgccggcggcgactagccaggccgaactccttaaactgattgat<br>caattgaataaagatcccaaaattcacggcatcctggtccaattaccattgccagaccatata<br>gatgagaagaaagtaattgatgcgattgccctggaaaaggatgttgacgggtttagcccggc<br>aaatgttggcaatctggttatcggcgataagtgtttctacccttgtactccccacggctgtatggtt<br>ttgttagaaaaggcaggtattgatcccaaaggtaagaaagcagtggtcgtcggccggagca<br>atatcgtcggcaaaccagtagcaatgatgcttcttgcacgtcacgccactgtgactatctgtca<br>ctctcgcactagggacctggcagccgaatgccggcaggccgacattctcattgcggccgta<br>gggaaaccggagttgattactggggatatgattaaagagggcgcggtagttatcgacgtagg<br>tattaatcgggtcggcgaaaagaaacttgtgggcgacgtccactttgagagtgcggcccaaa<br>aggcaggttggattactccagttccaggcggggtaggtcccatgactattgccatgctcttgaa<br>gaatactgttgaagcggcccggcgctaa
	gcvH	glycine cleavage system (gcv) H protein	Q2RH47 (*E.coli* codon optimized)	atgaatttcccggcacacctgcattattctaaggatcatgagtgggtagaagtcgatggtaatc<br>gtgcccgtatcggtattactgactacgcacaggagtcactgggtgacatagtgtttgtggaact<br>gccccaggtaggggatgaactggcgactggcgactcttttggtgtggtggagtctgtaaagtct<br>gcttctgacgtctatgcgccggtgggcggcaaagtggtggctgtgaacgaggccctcctgga<br>cgcgccgcaggacatcaacgcggatccctatggcaaaggctggatgatagaattagaattg<br>tctgatccgtcagaagttgaatctctcatggatgcctctgcttatctggaactggtaaaagaaga<br>gaaaggagaataa
	gcvL	gcv L protein	Q2RHM5 (*E.coli* codon optimized)	atgtcttatcagattgcaattattggcggtggccccggcggttatgtggcagcaattcgggcag<br>cacagctgggtgcaaaagtagtcgtaattgaacaggacgcactgggcggcacttgtctcaat<br>cgtggctgtattcccactaaagcactgctggcaggggcagcaatggtcaggggcattaaag<br>gggcagcagcatttggcattgatgtagaggattatcgggtagattatgcacgcctggcagcac<br>gtaaagacgcagtggtcaaacagcttactggaggcattgcatatttatttaagaagaataaag<br>tcgacctcataaaaggccgtgggttcctcaaaggccccggccagattgaagtggcaacagc<br>agatggcacgatcgagaacttacaagccgagaacatcatcctggcaacgggcagcgagc<br>cggcactgattaaagcactgggatataatggccgcacggtcgtgacttctactgaggcactg<br>gcatggactgaggtgccggcagagttactgattattggcggcggggtcattggctgtgagtttg<br>caactctctttgcaactctggggtctaaagtcactattgtcgaaatgatgcccgcaattttaccca<br>tgattgactctgagatttctaggcgcttttctatgctcctcaagaaaactggggtggaaatcaaa<br>actaaagcacagattactgaagtcaaagaagcaggcggccgggtccaggcaactcttgca<br>gacggccagactattaatgcagataaagttcttatttctattggccgccagtttaatactcgcgg<br>cttaggcctggaagatgcaggcattactctgggtcccaaaggtgaaattgtagttgacgaata<br>tctgcggacatctgtccccggcatttatgcaattggcgacgtgactaataaaattcagctggca<br>cacgtagcatctgcacagggcctggcagcagtgactactattatgggccggccgactaaagt<br>gaattatgacgcagtaccttcttgtatttatactctgccggagattgcaggcgtcgggttaacga<br>aggaagcagcagaagggcggggcatgaaagttagggtcggcaaatttccctttcaggcatc<br>tggcaaagccctgtgctctggcgagactgacggcatggtcaaaattattgcagaggcagagt<br>ctgaccgggtggttggggtctttattatgggtccgcacgcaactgaactcattgcagagggtgc<br>actggcagtcaataaaggaattaccgcaggcgaactggcagcaactattcacgcacatccc<br>actctgtctgaggcagttatggaggcagcagaggcagtacacgggctttctattcactcttag

gcvP	gcvP protein	Q2RH48 (*E.coli* codon optimized)	atgacttatattcccactacagcagcggagcagcagcagatgttagcagcatgtggtgcaca<br>ccggatggaagagctgttttctgacgtacctgcatctgtacgcctgggcagggaactcaatctc<br>ccacggccgatggctgaagcagaagtttggcgccacctggaggaattagcagggaagaat<br>aagaaactcgtctcttttctgggcgcaggagcttatgaacactatattccctctgtggtaggaca<br>cctcctggcacgctctgagttttatactgcatatactccctatcaaccggagatttctcaggga<br>ctctccaggcaatctttgaatttcaatctttaatttgtgagcttactggcctggatgtggcgactgc<br>atctcattatgacggggcaacagctatggcagaggcagcactggtagcatgtaatgcaactc<br>ggcggcagaaaattttggtctctcgctctgtgaatccccagtatcggactgtcctgtctacttatg<br>caaaaggtcagggtgtagaactggcagaggttccgttacaagatggtcggactgacctgga<br>ggcactggagaaactggcaggtaaagacgtcgcaggggtaattctgcaaaatcccaatttct<br>ttggtcagatcgaggcaatggcagaagcaactgatttggcccacaaagcaagggcattagg<br>tattgcagtcgtcgatcccgtttctcttggactgctggcagctccgggtgaatatggcgcagatct<br>ggcagtaggcgagggccagtctctgggtaatcctttgaactttggcggtccctacttgggttttat<br>tgcagcaagggagaaattagtgcggcgcttgccgggccgcattgtcggccagactaaaga<br>cgtggacggcaaacgcgcttatgtcctgactctgcaagcacgggagcagcatattcgccgg<br>gagaaagcaacttctaatatttgttctaatgaagctctgtgtgcactggcagcaactatttatcttg<br>cagcaatgggccgagagggcctgaaagaggtggcatctcagtgtctgttgaaagcacattat<br>gcacagaagaagctggctgcactaccaggggtaactccggtgtttaatggtcctttctttcatg<br>agtttgtgctgcaaactaaactttctccggcaactgtagcacggagactggcggaaaatggttt<br>tgcagcagggttcgacctgggtcgcttttatcccgaacttaagaatgcactgctctttactgtaac<br>tgaagtccggacaagggaggaaattgacgcactggtggcagcaatgaggggtattctggc<br>atga
gcvT	gcvT protein	Q2RH46 (optimized)	atggcagatttaaagaagacacccctgtacggtgaacacgtagcagcaggggcaaagatg<br>gttgaatttggcggctggttgatgcccgttcagtattcttctattattgaagaacatcagcgggttc<br>gtaattgcgctggcctctttgacgtttcacacatgggtgaaatcaccattaaaggacctgacgc<br>actggcactggttcagaaactgcttactaacgacgcagatcgggcaactggggatagagta<br>atatattctcctatgtgttatccggacggtggcgttgttgacgatctgctggtttatcctcgtggaga<br>aggggaatatctcctggttgttaacgcaggtaatattgataaagattttgcatggattcaggaga<br>acgcatctggttttcgggttgaggtttctaatatttctgctgcaactgcacaactggcactccagg<br>ggccacgagcactggaaattctccggccccttactagggttgatctggcatctctgggttattat<br>cgctggactgagggccaggttctgggcgttcattgtcttatttctcgcactggctatactggcga<br>agacggttttgagctttattttgaggcagcagcagcacctactatgtggcgtaatatactggctg<br>cgggcagggaggcaggcctggttccggcaggtttaggtgctcgtgatactcttaggctggag<br>gcagcactgcccctttacggccatgagttgggcccggatatttctccgctggaggcaggtttgc<br>atcgctttgttcgcctggaaaagggcgagttcaacgggagggaggcactggcagcacagc<br>gggaagcaggggttaggaggcaactggttggacttactatgattgatcggggcattccacgg<br>ccggaatatccggttctggcagcaggcaaagagattggttatgttacttctggttctctggcacc<br>aactctgggacaaaatattgcactggcattggttgcagcaggtactgtttctactggtggtgaa<br>gttgaagtatcaatacgcggccgtgttaatcgcgcacgggttgttaaactccccttttatcgccg<br>ccccaagaaataa
lipM	Octanoyltra nsferase	Q2RH52 (optimized)	atgccagcggaaacttggcgcctgctcgatactggcgtttctgatccttatactaatatggcaat<br>tgacgaggcaattctcctagagcaccgggagggaaagactccgcctactttacgtttctatgc<br>atggtctccgcccactatttctctgggttattttcagcagctagagaaagaaattgatctggagg<br>cagttaaagagcggggcctggggctggttcggcgcttaacaggcggccgggcagttctcca<br>tgacgatgaagttacttattctgttgttgcaagggaggatcatccactgatgattggtggcattcg<br>cccctcttacttaagattagcaaaagcactggcagcagggcttagagagctgggcgcaccg<br>gttgagattgcatctggccgtaaaggaggacgcgaggagcatactactgcggcatgttttgac<br>gcaccatcttggtatgagattacttgtggcggacgcaaacttgtaggttctgcacagactcgta<br>aaggcggcgttgttctccagcatggttctattgttctcactctgaatggcgacgatctctttgcagt<br>tctgaaaatgccctctgaggcagttcgccagcgcctcttggcaaaattttatcatcaggcatgc<br>ggtttagaggaagttctggggcggagggttgaggcaggggttattaaagaaaatattgttcgg<br>gcatttactaggctctatgcagttgaatttgttcctggcggtctgactgagggagagaaagggc<br>gccttaaagaactgcgggcaaaatatgcagcagcagactggctcaaaagacggtga

	shmt	Serine hydroxymethyltransferase	Q2RFW7 (optimized)	atgaatctggaaactgtagcaaaagttgatcccgaaattgtagcagcagtcaggggcgagc<br>ttcaacgccagcggactcacctggaactaattgcatctgagaattttgtctctcaggcagtgat<br>ggaggcgtattcttgtgtcctgactaataaatatgcagaagggtatcccggcaaacgctattat<br>ggcggttgtgaatgggcagacgtagtcgaaaatttggcacgcgaaagggcaaaagcactc<br>tttggggcagaacacgcaaatgtccagccccattctggctctcaggcaaacacggcggtcta<br>tctggcagttctgaatcctggcgataaagcactgggcatgaatctggcacacggcggccattt<br>gactcacggttctccggtatctctttctggcaaatattataatttctgcttctatggcgtcgatgcaa<br>agactggccgtattgactatgacgcagtagcacgcattgcacgtgaggagcggcccagact<br>gattgtcgcaggggcatctgcatatccgcgtgtaattgactttgcacgttttcgcgagattgcag<br>acgaggtcggcgcactgctgatggtggatatggcacatattgcaggactggtggcagcaggt<br>attcatcccaatccggtaccctatgcacattttgtgaccactactactcataaaactatgcgcgg<br>tcctcgtggtggaataattctgactactagggaatatgcacgcgatattgataaagcagtctttc<br>ccggtgtccagggcgggcccctgatgcacgtcatcgctgcgaaagccgtcgcattaaaaga<br>ggccatgctcccggagttcaagcgttatcaggagcaaattgttactaatgcacgtactctggc<br>agacgcactcatgggctatggatttaatctggtttctggcggtactgacaatcacctaatgctcg<br>tcgacctacgcaataagaacattactggccgggaggcagaagatattctggcatctgtccag<br>attactgtcaataagaacgcaattccctttgacccgcagaaaccctctgtaacttctggcattcg<br>cctgggaacggcagcactgacttctcgcggcatggacgcagacgcaatggtccaggtggc<br>gcgagcaattgacctggcattatcttatggtccggatgagaagaagctggaggaagcaagg<br>ggcatcgtcgcagaactctgtcgggcatttcctctctatcaggagttagactaa
Pseudomonas stutzeri	*ptdh**	Phosphite dehydrogenase mutant	17X-PTDH[2] Mutations: D13E, M26I, V71I, E130K, Q132R, Q137R, I150F, Q215L, R275Q, L276Q, I313L, V315A, A319E, A325V, E332N, C336D. E175A	atgctgccgaaactcgttataactcaccgagtacacgaagagatcctgcaactgctggcgcc<br>acattgcgagctgataaccaaccagaccgacagcacgctgacgcgcgaggaaattctgcg<br>ccgctgtcgcgatgctcaggcgatgatggcgttcatgcccgatcgggtcgatgcagactttctt<br>caagcctgccctgagctgcgtgtaatcggctgcgcgctcaagggcttcgacaatttcgatgtg<br>gacgcctgtactgcccgcggggtctggctgaccttcgtgcctgatctgttgacggtcccgactg<br>ccgagctggcgatcggactggcggtggggctggggaggcatctgagggcagcagatgcgt<br>tcgtccgctctggcaagttccggggctggcaaccacggttctacggcacggggctggataac<br>gctacggtcggcttccttggcatgggcgccatcggactggccatggctgatcgcttgcaggga<br>tggggcgcgaccctgcagtaccacgcggcgaaggctctggatacacaaaccgagcaacg<br>gctcggcctgcgccaggtggcgtgcagcgaactcttcgccagctcggacttcatcctgctggc<br>gcttcccttgaatgccgataccctgcatctggtcaacgccgagctgcttgccctcgtacggccg<br>ggcgctctgcttgtaaacccctgtcgtggctcggtagtggatgaagccgccgtgctcgcggcg<br>cttgagcgaggccagctaggagggtatgcggcggatgtattcgaaatggaagactgggctc<br>gcgcggacaggccacagcagatcgatcctgcgctgctcgcgcatccgaatacgctgttcact<br>ccgcacatagggtcggcagtgcgcgcggtgcgactggagattgaacgttgtgcagcgcag<br>aacatcctccaggcattggcaggtgagcgcccaatcaacgctgtgaaccgtctgcccaagg<br>ccgagcctgccgcatgttga

Table S11: Table of commercial reagent prices used in cost analysis. Data found in Ref. 1

Chemical	Vendor	Cost
Cell lysate-based TX/TL	[3]	\$90 / L
THF	Millipore Sigma	\$970 / g
ATP	Millipore Sigma	\$33.6 / g
NADH	Cayman Chemicals	\$60 / g
NADPH	Santa Cruz Biotechnology	\$428 / g
Na_2HPO_3	Millipore Sigma	\$118 / kg
Serine	Chem-Impex International	\$980 / 5 kg
Glycine	Chem-Impex International	\$80 / 5 kg

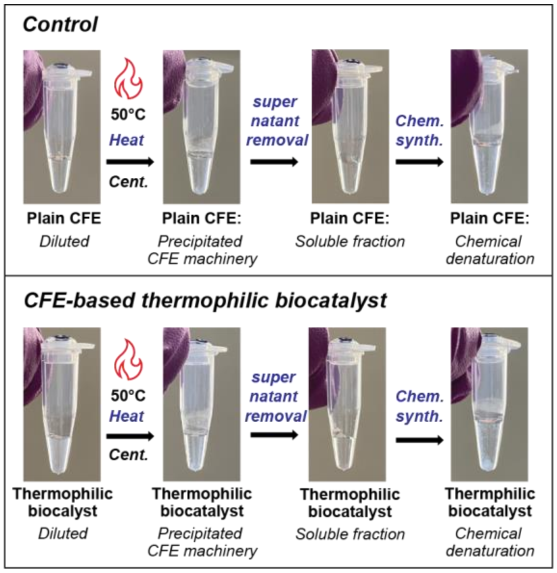

Figure S1: Images of Plain CFE and the CFE-based thermophilic Module 1 biocatalyst during different steps of the bioprocess workflow. Plain CFE: CFE without expressing thermophilic pathway genes. Module 1: THF-dependent formate fixation. Plain CFE was incubated for 16 hours to simulate the gene expression step in the thermophilic biocatalyst. After dilution, plain CFE and the thermophilic biocatalyst were heat denatured and a pellet can be observed in both cases (precipitated CFE machinery). The proteins in the soluble fraction (supernatant) are taken for chemical synthesis. The supernatant is chemically denatured. The pellet from the thermophilic biocatalyst is much larger than that of the control supporting the expression of thermophilic genes.

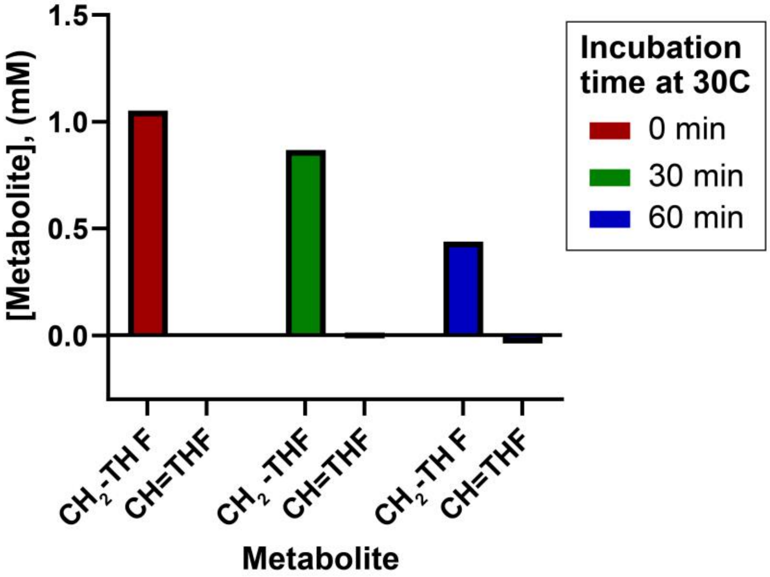

Figure S2: Stability of CH_2-THF at 30°C over time**.** 1mM commercial CH_2-THF was incubated in 50µL Tris buffer at 30°C for the specified time. After incubation, 50 µl of 5% acetic acid in methanol spiked with 0.1 mM catechol (internal standard) was added and the reaction was directly analyzed via LC/MS. CH=THF data shown to confirm decrease in CH_2-THF concentration is due to temperature degradation as opposed to reversion to CH=THF.

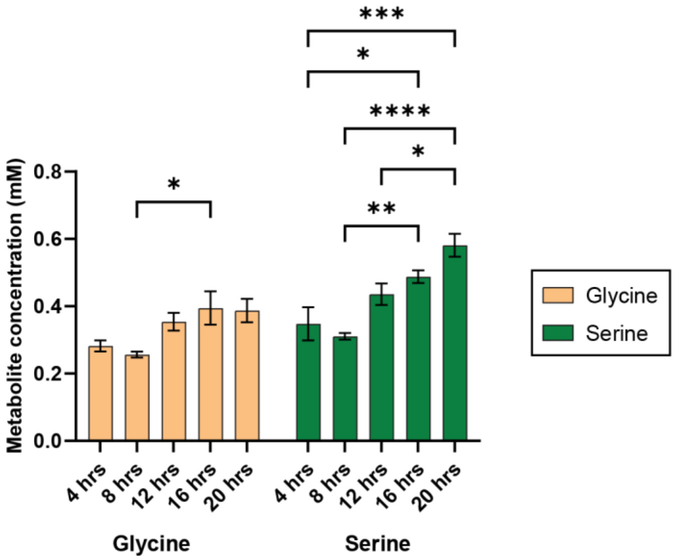

Figure S3: Extended comparisons between groups shown in Figure 4D. Metabolite as a function of gene expression time. Data points represent mean ± SEM, n = 3, with *p < 0.05, **p < 0.005, ***p < 0.001, ****p < 0.0001. Data were analyzed using a two-way ANOVA followed by a multiple comparisons test via the Tukey method.

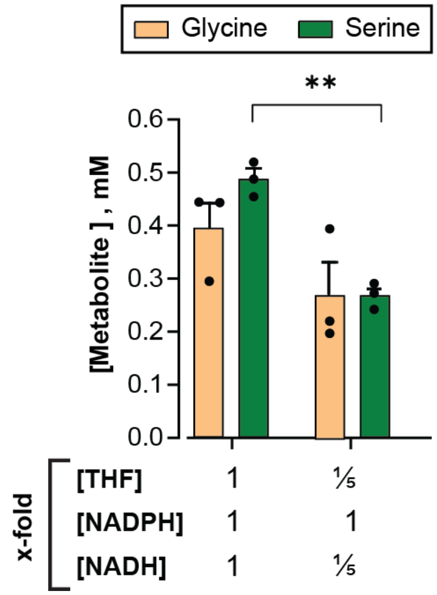

Figure S4: Concentration of glycine/serine from formate as a function of simultaneous reduction in THF and NADH concentrations. Base case cofactor concentration: 2mM THF, 2mM NADPH, 2mM NADH, 2mM ATP(1x-fold [cofactor]: 2mM THF, 2mM NADPH, 2mM NADH, 2mM ATP). Reaction conditions: Gene expression: 30°C, 16 hours; Biocatalyst dilution, 10x; Heat denaturation: 50°C, 10 minutes; Chemical synthesis: 30°C, 4 hours; Substrate/cofactor supplementation, 2 mM CH_2O_2, 1 mM H_2CO_3, 1 mM NH_3, 2 mM ATP, 5 mM Na_2HPO_3 or the specified cofactor concentration. Bars represent mean ± standard error of the mean (SEM), n = 3, $^{**}p < 0.005$. Data were analyzed using two-way ANOVA followed by a multiple comparisons test via the Tukey method.

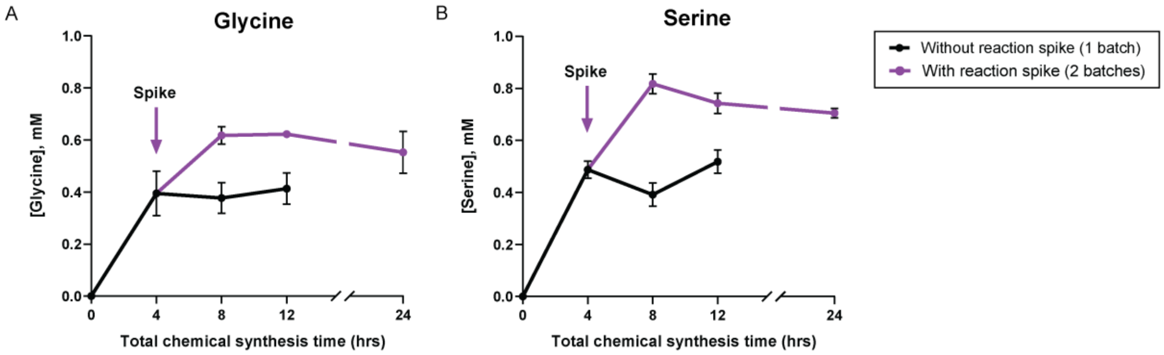

Figure S5: Individual glycine and serine concentrations shown in Figure 6B. Glycine and serine concentrations as a function of chemical synthesis time used to calculate total amino acid yields shown in Figure 6B. Concentrations shown as a result of spiking reactions with chemicals and cofactors to initiate a 2nd batch at the 4-hour point (purple: with reaction spike) and when chemicals and cofactors are not spiked (black: without reaction spike, data from Figure 2A). Data points represent mean ± SEM, n = 3.

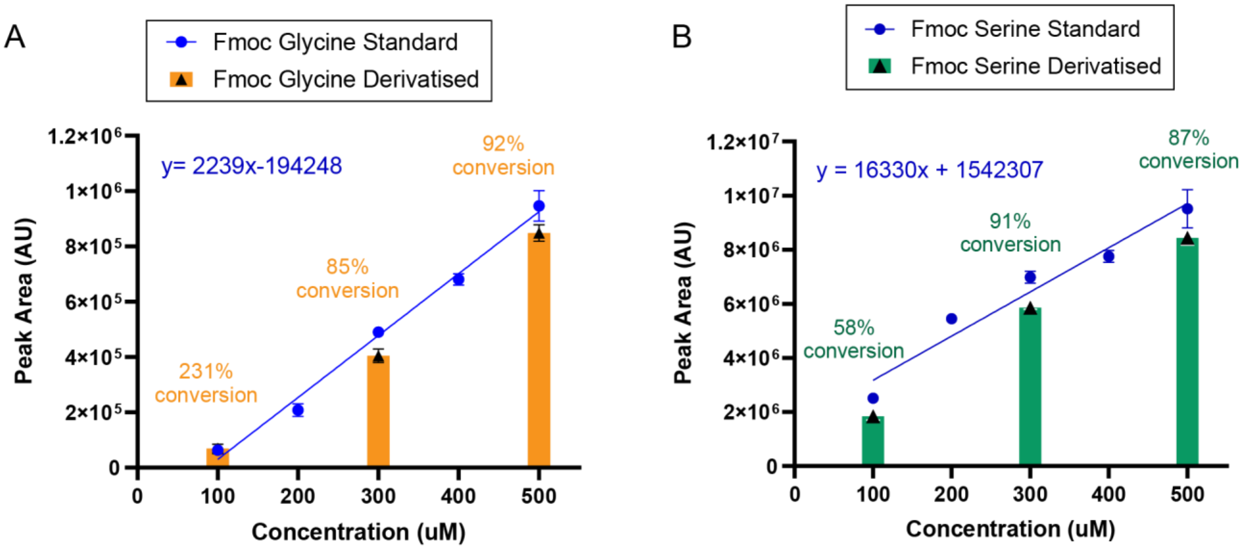

Figure S6: Efficiency of derivatization of glycine and serine into Fmoc=Gly and Fmoc-Ser. The line graphs show peak areas for commercial Fmoc-glycine (left) and commercial Fmoc-serine (right). The bar graphs show peak areas for commercial glycine (left, orange) and commercial serine (right, green) derivatised with Fmoc-Cl as described in the methods section. Data points represent mean ± SEM, n = 3.

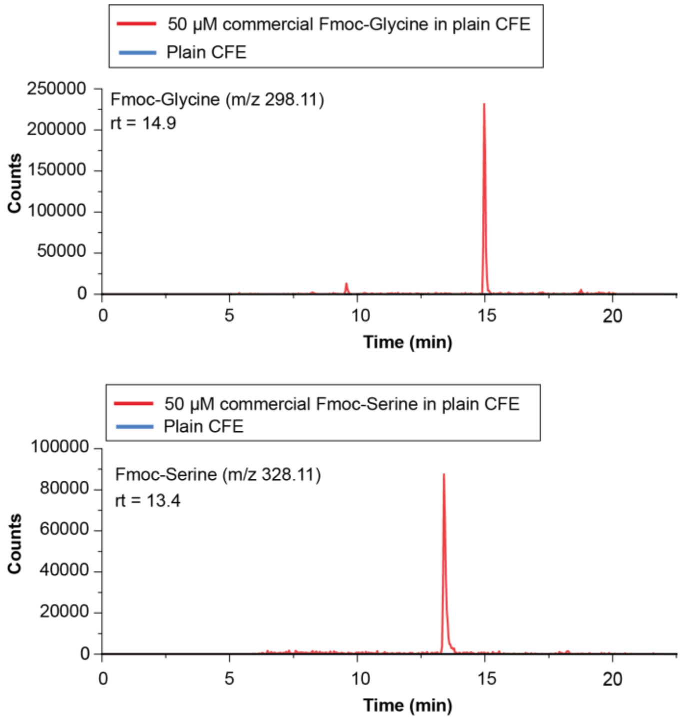

Figure S7: LC/MS traces of commercial Fmoc-Serine and Fmoc-glycine in plain cell-free expression (CFE)**.** Chemicals were identified via extracted ion chromatogram at the m/z specified. rt= retention time. Figure obtained from Chowdhury et al [1].

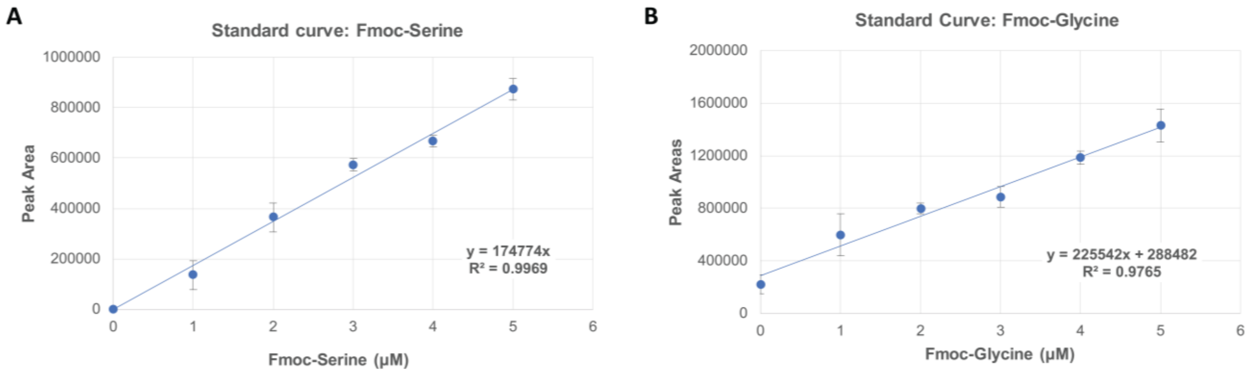

Figure S8. Standard curves of Fmoc-Serine and Fmoc-Glycine using Fmoc derivatized serine and glycine under the bioprocess workflow conditions. Traces and standard curves for Fmoc-Serine and Fmoc-Glycine were generated by adding TX/TL (75% vol) and water to reach 5 µL in a PCR tube, then adding the specified chemical concentration and Tris buffer up to 50µl, then denaturing the CFE proteins by adding 50µl of 5% acetic acid in methanol spiked with 2 mM Boc-Serine (internal standard). The reactions were then derivatized following the Amino Acid Derivatization protocol described in the text. The specified chemical concentration is the concentration in the final 200µl reaction volume. Figure from Chowdhury et al [1].

.

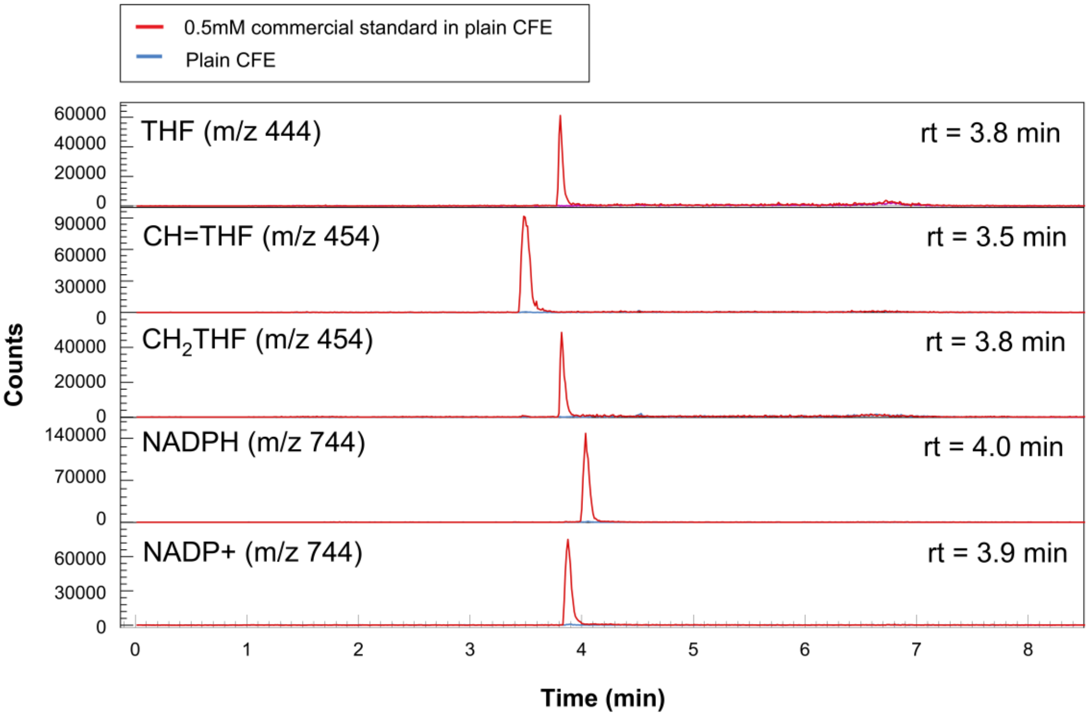

Figure S9: LC/MS traces of commercial tetrahydrofolate (THF), 5,10-methenyltetrahydrofolate (CH=THF), 5,10 methyleneltetrahydrofolate (CH_2-THF), NADPH, and NADP+ in plain cell-free expression (CFE). Chemicals were identified via extracted ion chromatogram at the m/z specified. rt = retention time.

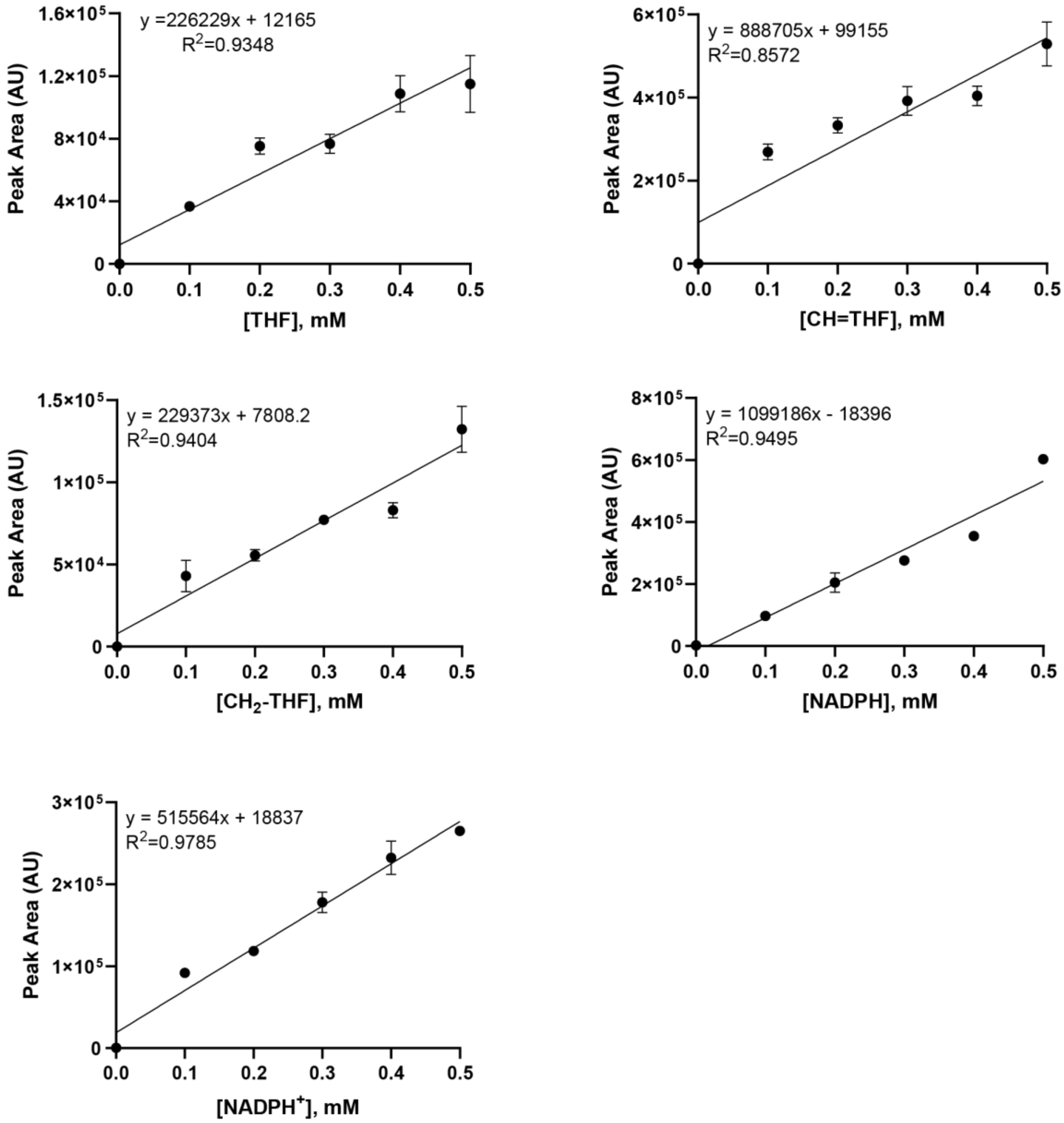

Figure S10: Standard curves using commercial tetrahydrofolate (THF), 5,10-methenyltetrahydrofolate (CH=THF) and 5,10 methylenetetrahydrofolate (CH_2-THF), NADPH and $NADP^+$. Traces and standard curves for THF, CH=THF, CH_2-THF, NADPH, and $NADP^+$ were generated by adding TX/TL (75% vol) and water to reach 5 µL in a PCR tube, then adding the specified chemical concentration and Tris buffer up 50µl, then denaturing the CFE proteins by adding 50µl of 5% acetic acid in methanol spiked with 0.1 mM catechol (internal standard). The specified chemical concentration is the concentration in the 100µl total reaction volume. Denatured reactions were centrifuged at 3,600x*g* for 10 min and the supernatant directly analyzed.

References

1. Chowdhury, S. *et al.* Carbon Negative Synthesis of Amino Acids Using a Cell-Free-Based Biocatalyst. *ACS Synth Biol* **13**, 3961-3975 (2024).

2. Zhou et al. Crystal structures of phosphite dehydrogenase provide insights into nicotinamide cofactor regeneration. Biochemistry, 51, 4263-4270 (2012)

3. Rasor, B. J. *et al.* Toward sustainable, cell-free biomanufacturing, *Current Opinion in Biotechnology* , **69**, 136-144 (2021).
